# Medical termination for pregnancy in early first trimester (≤ 63 days) using combination of mifepristone and misoprostol or misoprostol alone: a systematic review

**DOI:** 10.1186/s12905-020-01003-8

**Published:** 2020-07-07

**Authors:** Ferid A. Abubeker, Antonella Lavelanet, Maria I. Rodriguez, Caron Kim

**Affiliations:** 1 UNDP/UNFPA/UNICEF/WHO/World Bank Special Programme of Research, Development and Research Training in Human Reproduction (HRP), Department of Reproductive Health and Research, World Health Organization, Geneva, Switzerland; 2 Department of Obstetrics & Gynecology, Oregon Health & Science University, Oregon, Portland USA

**Keywords:** Medical abortion, First trimester, Mifepristone, Misoprostol, Systematic review

## Abstract

**Background:**

A wide range of drugs have been studied for first trimester medical abortion. Studies evaluating different regimens, including combination mifepristone and misoprostol and misoprostol alone regimens, show varying results related to safety, efficacy and other outcomes. Thus, the objectives of this systematic review were to compare the safety, effectiveness and acceptability of medical abortion and to compare medical with surgical methods of abortion ≤63 days of gestation.

**Methods:**

Pubmed and EMBASE were systematically searched from database inception through January 2019 using a combination of MeSH, keywords and text words.

Randomized controlled trials on induced abortion at ≤63 days that compared different regimens of medical abortion using mifepristone and/or misoprostol and trials that compared medical with surgical methods of abortion were included.

We extracted data into a pre-designed form, calculated effect estimates, and performed meta-analyses where possible. The primary outcomes were ongoing pregnancy and successful abortion.

**Results:**

Thirty-three studies composed of 22,275 participants were included in this review. Combined regimens using mifepristone and misoprostol had lower rates of ongoing pregnancy, higher rates of successful abortion and satisfaction compared to misoprostol only regimens. In combined regimens, misoprostol 800 μg was more effective than 400 μg. There was no significant difference in dosing intervals between mifepristone and misoprostol and routes of misoprostol administration in combination or misoprostol alone regimens. The rate of serious adverse events was generally low.

**Conclusion:**

In this systematic review, we find that medical methods of abortion utilizing combination mifepristone and misoprostol or misoprostol alone are effective, safe and acceptable. More robust studies evaluating both the different combination and misoprostol alone regimens are needed to strengthen existing evidence as well as assess patient perspectives towards a particular regimen.

## Background

Medical methods emerged as an alternative to surgical abortion with the discovery of prostaglandins in the early 1970s [1–3]. Their use has evolved in the last two decades and various drugs have been used for first trimester medical abortion. Several studies have explored utilization of mifepristone, methotrexate and various prostaglandins with different doses, routes and intervals of administration [4]. A Cochrane review compared different medical methods for first trimester abortion in 2011 and since that time, there has been growing evidence assessing the effectiveness and safety of medical methods using two specific regimens: the combination regimen (mifepristone and misoprostol) and misoprostol alone [5].

However, individual studies evaluating medical management of abortion at ≤63 days have not demonstrated superiority of one of these regimens. Not only have studies compared combination of mifepristone and misoprostol (combination mifepristone misoprostol) with misoprostol alone [6–8], other studies have looked at different routes and doses of misoprostol in combined regimens [9, 10], besides comparing different intervals between mifepristone and misoprostol doses [11–13]. Similarly, different misoprostol only regimens have been evaluated [14].

The 2012 World Health Organization (WHO) safe abortion guideline had varying regimens for induced abortion at < 12 weeks. With the emergence of new evidence, this systematic review was done as part of the evidence synthesis for the WHO guidance on medical abortion. Options for medical abortion vary globally, and evidence-based guidance is needed to inform clinical care in selecting a regimen. The objectives of this review were to compare the effectiveness, safety and acceptability of different regimens of medical abortion containing mifepristone and/or misoprostol and to compare medical with surgical methods of abortion at ≤63 days of gestational age.

## Methods

### Search strategy

We searched Pubmed and EMBASE for randomized controlled trials on induced abortion at ≤63 days. Our search was from database inception through January 2019 using a combination of MeSH, keywords and text words (Additional file 1).

### Selection criteria

Inclusion criteria comprised randomized controlled trials (RCTs) that compared different medication regimens for induced abortion at ≤63 days using mifepristone and/or misoprostol; different frequencies of administration of misoprostol in combination regimens; different doses and dosing intervals of misoprostol in combination regimens; different routes of misoprostol in combination regimens; and different dosing regimens and routes in misoprostol only regimens. We also included trials that compared surgical and medical abortion using combination or misoprostol alone regimens. We excluded studies that included induced abortion > 63 days, missed abortion, miscarriage, fetal demise and those that did not report on the primary outcomes. We also excluded studies comparing medical regimens beyond mifepristone and/or misoprostol, such as those using methotrexate or gemeprost. In addition, we excluded studies that compared various mifepristone dosages beyond the WHO recommended 200 mg dose, as a previously conducted Cochrane review showed effectiveness of mifepristone at this lower dose (5).

All search results (titles, abstracts and when necessary, full articles) were screened using the Covidence tool [15].

### Data extraction and analysis

Data extraction was performed using a standardized data-abstraction form.

The primary outcomes were ongoing pregnancy and successful abortion (defined as uterine evacuation without need for surgical intervention). Secondary outcomes were: safety (defined as serious adverse events and complications; such as hospitalization; blood transfusion; need for surgical interventions beyond uterine evacuation; or death), expulsion time from initiation of treatment, side effects (including bleeding; pain; and vomiting) and satisfaction.

For dichotomous data (e.g., complete abortion rate), we used the number of events in the control and intervention groups of each study to calculate Risk Ratios (RRs) with 95% confidence intervals for our primary outcome, and secondary outcomes as available. Analyses were conducted using RevMan version 5.3 (Copenhagen, Denmark: The Nordic Cochrane Centre, The Cochrane Collaboration, 2014).

We used GRADEpro software and Cochrane methods to evaluate the overall quality of the body of evidence for the main review outcomes. We relied on GRADE (Grading of Recommendations, Assessment, Development and Evaluations) criteria (e.g., risk of bias, consistency of effect, imprecision, indirectness, and publication bias) to assess the quality of the evidence. The Cochrane Risk of Bias Assessment tool was used to assess risk of bias across studies [16]. We specifically assessed: selection (random sequence generation and allocation concealment); performance (blinding of participants and personnel); detection (blinding of outcome assessors); attrition (incomplete outcome data); reporting (selective reporting); and other biases. Studies were ranked as low risk, high risk, or unclear risk using the criteria outlined by the *Cochrane Handbook for Systematic Reviews of Interventions* [16].

Two review authors (FAA and CK) independently performed study selection, data extraction, assessment of risk of bias and quality of evidence. Discrepancies were resolved by discussion with the third author (MIR).

## Results

**Fig. 1:**
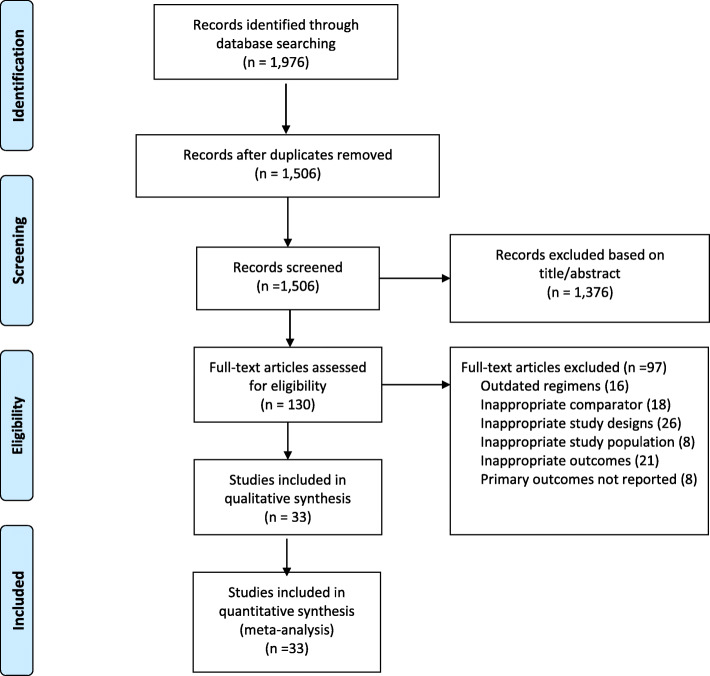
PRISMA flow diagram

The initial search yielded 1506 articles, of which 33 articles fit our inclusion criteria (Fig. 1). Studies included for this review were conducted across 19 countries with a total of 22,275 participants. Using the World Bank’s 2018 classification of economies, the articles represent data from six high income economies, six upper-middle income economies, six lower-middle income economies and one low income economy [17]. The year of publication ranged from 1994 to 2017. The characteristics of the included studies are shown in Table 1. Approximately 85% of the included studies had a low risk of selection bias based on random sequence generation and 78% had a high risk of performance bias (Additional file 2).

**Table 1:** Characteristics of included studies

S.No	Author, year	Methods	Setting	Participants	Interventions
1.	Blanchard et al. 2005 [14]	Randomized controlled trial	KEM Hospital in Pune, India, and Hungvuong Hospital in Ho Chi Minh City, Vietnam.	Women seeking pregnancy termination at 56 days or less of amenorrhea. All eligible women had a transvaginal ultrasound scan to confirm duration of pregnancy.	Misoprostol oral 400 μg every 3 h for 4 doses (*N* = 36) vs. Misoprostol oral 800 μg every 6 h for 2 doses (*N* = 24) vs. Misoprostol vaginal 600 μg for 1 dose (*N* = 40)
2.	Blum et al. 2012 [6]	Randomized controlled trial	Two large maternity hospitals: the Centre de Maternite et Neonatologie de la Rabta in Tunisia (*n* = 193) and Hung Vuong Hospital, Ho Chi Minh City, Vietnam (*n* = 248).	Pregnant women presenting for early medical abortion up to 63 days since their last menstrual period.	Mifepristone + misoprostol combined Mifepristone 200 mg on day 1 and 800 μg buccal misoprostol followed by placebo 3 h later on day 2 (*N* = 220) vs. Misoprostol alone Placebo on day 1 and 1600 μg of misoprostol (2 doses of 800 μg, given 3 h apart) on day 2 (*N* = 221)
3.	Chai et al. 2013 [18]	Randomized controlled trial	Conducted at the Family Planning Association in Hong Kong.	Healthy women aged 18 years or older who requested termination of pregnancy of up to 63 days’ gestation. A transvaginal ultrasound examination was performed to verify the duration of pregnancy and to determine the gestational age.	Misoprostol buccal Misoprostol buccal 800 μg (four 200 μg misoprostol buccal and four sublingual placebo) 48 h after receiving mifepristone (*N* = 45) vs. Misoprostol sublingual Misoprostol sublingual 800 μg (four 200 μg misoprostol sublingual and four buccal placebo) 48 h after receiving mifepristone (N = 45)
4.	Chawdhary et al. 2009 [19]	Randomized controlled trial	Department of Obstetrics and Gynecology, Tribhuvan University Teaching Hospital, Kathmandu, Nepal.	Trans vaginal ultrasound demonstrating an intact single intrauterine pregnancy up to a 63-day period of gestation.	Mifepristone + misoprostol combined Mifepristone 200 mg on day 1 and vaginal misoprostol 800 μg on day 3 (*N* = 50) vs. Misoprostol alone Misoprostol vaginal (800 μg) on day 1 and 3 (total dose 1600 μg) (*N* = 50)
5.	Chong et al. 2012 [9]	Randomized controlled trial	Three clinics in the Republic of Georgia and at Hoc Mon Hospital in Vietnam.	Women who presented for termination of pregnancy with gestations up to 63 days since last menstrual period (LMP).	Misoprostol buccal 400 μg Misoprostol buccal 400 μg (two 200 μg misoprostol and two placebo pills) 36–48 h after mifepristone (*N* = 559) vs. Misoprostol buccal 800 μg Misoprostol buccal 800 μg (four 200 μg misoprostol pills) 36–48 h after mifepristone (*N* = 563)
6.	Coyaji et al. 2007 [20]	Randomized controlled trial	K.E.M. Hospital in Pune (*n* = 150) and the Health Centre, Larsen and Toubro Limited, Mumbai, India (*n* = 150).	Women seeking termination of pregnancies could participate if they had amenorrhoea of 8 weeks or less.	Two doses of misoprostol Two doses of 400 μg oral misoprostol taken in 3 h interval 48 h after mifepristone (*N* = 150) vs. Single dose of misoprostol Single dose of 400 μg oral misoprostol and 2 placebo tablets 3 h later 48 h after mifepristone (N = 150)
7.	Creinin et al. 2007 [12]	Randomized controlled trial	Four centers: The University of Pittsburgh, Oregon Health and Science University, Northwestern University, and the University of Southern California. The University of Pittsburgh served as the sponsoring institution.	Healthy women requesting an elective abortion, had an intrauterine pregnancy less than or equal to 63 days of gestation on the day of mifepristone administration as confirmed by vaginal ultrasound.	Misoprostol 800 μg vaginal immediately after taking mifepristone (*N* = 567) vs. Misoprostol 800 μg vaginal misoprostol 24 h after taking mifepristone (*N* = 561)
8.	Dahiya et al. 2011 [21]	Randomized controlled trial	Postpartum center at PGIMS Rohtak, India.	Healthy women with intrauterine pregnancy < 56 days based on menstrual history and clinical examination.	Misoprostol oral 400 μg 24 h after mifepristone (*N* = 48) vs. Misoprostol sublingual 400 μg 24 h after mifepristone (N = 45)
9.	Dahiya et al. 2012 [7]	Randomized controlled trial	Outpatient department of Obstetrics and Gynecology of Pt BDSharma PGIMS, Rohtak, India.	Women with amenorrhea < 56 days, age > 18 years, request for elective abortion with the indication as per the guidelines of the 1971 MTP act.	Mifepristone + misoprostol combined Mifepristone 200 mg oral and 800 μg buccal misoprostol after 24 h (N = 50) vs. Misoprostol alone Misoprostol buccal 800 μg (N = 50)
10.	El-Refaey et al. 1994 [22]	Randomized controlled trial	Department of Obstetrics and Gynaecology, University of Aberdeen	Women requesting termination of pregnancy of less than 56 days amenorrhea confirmed by ultrasound scan examination and fulfilling the criteria of the 1967 Abortion Act.	Misoprostol oral 800 μg single dose 36–48 h after mifepristone (*N* = 75) vs. Misoprostol oral 400 μg repeated 2 h later 36–48 h after mifepristone (N = 75)
11.	El-Refaey et al. 1995 [23]	Randomized controlled trial	Fertility-control clinic, Aberdeen Royal Hospitals, Aberdeen, Scotland.	Women requesting termination of pregnancy within 63 days from the onset of amenorrhea and fulfilling the criteria of the 1967 Abortion Act.	Misoprostol oral 800 μg 36–48 h after mifepristone (*N* = 130) vs. Misoprostol vaginal 800 μg 36–48 h after mifepristone (*N* = 133)
12.	Fekih et al. 2010 [24]	Randomized controlled trial	Department of Obstetrics and Gynecology in Farhat Hached Teaching Hospital, Sousse, Tunisia.	Women requesting 1st trimester abortion of less than or equal to 56 days from their last menstrual period, determined by vaginal probe ultrasound and a maximum embryonic length of 17 mm.	Mifepristone + misoprostol combined Mifepristone 200 mg followed by 400 μg of oral misoprostol after 48 h (*N* = 126) vs. Misoprostol alone Misoprostol sublingual 800 μg (repeated every 4 h for up to a maximum of 3 doses) (N = 126)
13.	Goel et al. 2011 [25]	Randomized controlled trial	Obstetrics and Gynaecology Department, MMIMSR, Mullana (Ambala), Haryana, India.	Healthy pregnant women, who were requesting an elective abortion and had a single intrauterine pregnancy of < 7 weeks (49 days) of gestation as confirmed by transvaginal ultrasonography.	Misoprostol vaginal 400 μg simultaneously with mifepristone (*N* = 40) vs. Misoprostol vaginal 400 μg 24 h after mifepristone (N = 40)
14.	Guest et al. 2007 [11]	Randomized controlled trial	Ninewells Hospital, Dundee, Scotland.	An intrauterine pregnancy confirmed on pelvic ultrasound scan, gestation not exceeding 63 days at the administration of mifepristone and participants must be aged 16 years or older, seeking a termination of pregnancy.	Misoprostol vaginal 800 μg after 6 h of mifepristone (*N* = 225) vs. Misoprostol vaginal 800 μg after 36–48 h of mifepristone (N = 225)
15.	Hamoda et al. 2005 [26]	Randomized controlled trial	Aberdeen Royal Infirmary, United Kingdom.	Women with a viable singleton intrauterine pregnancy (confirmed by transvaginal ultrasound scan) requesting medical abortion up to 13 weeks of gestation. Data aggregated by gestational age.	Misoprostol sublingual 600 μg followed 3 h later by a further dose of 400 μg sublingual misoprostol (*N* = 57) vs. Misoprostol vaginal 800 μg followed 3 h later by a further dose of 400 μg vaginal misoprostol (*N* = 72)
16.	Jain et al. 2002 [27]	Randomized controlled trial	Women’s and Children’s Hospital and affiliated clinics, Los Angeles County-University of Southern California Medical Center and San Francisco General Hospital, University of California, San Francisco, United States.	A total of 250 healthy women desiring termination of pregnancies < 56 days gestation were enrolled.	Mifepristone + misoprostol combined Mifepristone 200 mg followed after 48 h by 800 μg of vaginal misoprostol (repeated every 24 h for up to a maximum of 3 doses) (*N* = 125) vs. Misoprostol alone Placebo on day 1 and misoprostol vaginal 800 μg repeated every 24 h for up to a maximum of 3 doses (N = 125)
17.	Middleton et al. 2005 [28]	Randomized controlled trial	Two sites in Rochester, NY, United States.	Women seeking abortion with pregnancies through 56 days LMP.	Misoprostol buccal 800 μg 1–2 days after mifepristone (*N* = 223) vs. Misoprostol vaginal 800 μg 1–2 days after mifepristone (*N* = 219)
18.	Ngoc et al. 2011 [8]	Randomized controlled trial	Tertiary hospital in Ho Chi Minh City, Vietnam.	Women with gestational age up to 63 days by LMP, living and working within an hour from the hospital desiring medical abortion.	Mifepristone + misoprostol combined Mifepristone 200 mg followed 24 h later by 800 μg buccal misoprostol followed by placebo 24 h later after misoprostol (*N* = 202) vs. Misoprostol alone Placebo followed by 800 μg buccal misoprostol repeated 24 and 48 h later (1600 μg total) (*N* = 198)
19.	Prasad et al. 2009 [29]	Randomized controlled trial	Department of Obstetrics and Gynecology, Maulana Azad Medical College, New Delhi, India.	Women with gestational age up to 49 days confirmed by clinical examination and pelvic ultrasound seeking abortion.	Medical abortion-misoprostol vaginal 800 μg (*N* = 70) vs. Surgical intervention (N = 70)
20.	Raghavan et al. 2009 [30]	Randomized controlled trial	University Clinic, Municipal Clinical Hospital No.1, Chisinau, the Republic of Moldova.	The date of onset of last menses plus pelvic examination were used to calculate gestational age, with ultrasound confirmation as needed.	Misoprostol sublingual 400 μg 24 h after mifepristone (*N* = 240) vs. Misoprostol oral 400 μg 24 h after mifepristone (N = 240)
21.	Raghavan et al. 2010 [31]	Randomized controlled trial	University Clinic, Municipal Clinical Hospital No.1, Chisinau, the Republic of Moldova.	Women with gestational age through 63 days by LMP presenting for abortion. Gestational age was determined by one or more assessment method: last menses method, pelvic examination and ultrasound.	Misoprostol buccal 400 μg 24 h after mifepristone (*N* = 277) vs. Misoprostol sublingual 400 μg 24 h after mifepristone (*N* = 273)
22.	Schaff et al. 2000 [32]	Randomized controlled trial	Sixteen US primary care and referral abortion facilities.	Participants were at least 18 years old, no more than 56 days pregnant, healthy and desired an abortion.	1) Misoprostol vaginal 800 μg 1 day later after mifepristone (*N* = 745) vs. 2) Misoprostol vaginal 800 μg 2 days later after mifepristone (*N* = 778) vs. 3) Misoprostol vaginal 800 μg 3 days later after mifepristone (*N* = 772)
23.	Schaff et al. 2001 [33]	Randomized controlled trial	Multicenter study at 15 sites in United States.	Women no more than 63 days pregnant, confirmed by sonogram, desiring an abortion.	Misoprostol oral 800 μg 24 h after mifepristone and 400 μg, then another 400 μg misoprostol 2 h later, last dose no later than midnight on day 2 (*N* = 548) vs. Misoprostol vaginal 800 μg 24 h after mifepristone (*N* = 596)
24.	Schaff et al. 2002 [34]	Randomized controlled trial	Multicenter study at 14 sites in United States	Women no more than 63 days pregnant, confirmed by sonogram, desiring an abortion.	1) Misoprostol oral 400 μg 48 h after mifepristone (*N* = 220) vs. 2) Misoprostol oral 800 μg 48 h after mifepristone (*N* = 269) vs. 3) Misoprostol vaginal 800 μg 48 h after mifepristone (*N* = 522)
25.	Shannon et al. 2006 [35]	Randomized controlled trial	Three clinics associated with major research universities in Canada; two in major urban areas and one in a periurban area.	Women aged 16 years or older, seeking elective abortion of pregnancies less than 56 days since last menstrual period or on vaginal ultrasound.	1) Misoprostol oral 400 μg 24–48 h after mifepristone (*N* = 319) vs. 2) Misoprostol oral 600 μg 24–48 h after mifepristone (N = 319) vs. 3) Misoprostol vaginal 800 μg 24–48 h after mifepristone (*N* = 318)
26.	Tang et al. 2003 [36]	Randomized controlled trial	Department of Obstetrics and Gynaecology, The University of Hong Kong, Queen Mary Hospital, Hong Kong SAR, China.	Women with gestational age of less than 9 weeks, confirmed by US, requesting legal termination of pregnancy.	Misoprostol sublingual Misoprostol sublingual 800 μg (and four tablets of vaginal placebo) 48 h after receiving mifepristone (*N* = 112) vs. Misoprostol vaginal Misoprostol vaginal 800 μg (and four tablets of sublingual placebo) 48 h after receiving mifepristone (N = 112)
27.	Tendler et al. 2015 [37]	Randomized controlled trial	Department of Obstetrics and Gynecology, Galilee Medical Center, Nahariya, Israel.	Women no more than 55 days gestational age desiring medical abortion.	Misoprostol oral 400 μg 2 h after mifepristone (*N* = 50) vs. Misoprostol oral 400 μg 48 h after mifepristone (N = 50)
28.	Verma et al. 2011 [13]	Randomized controlled trial	Department of Obstetrics and Gynaecology, Hind Institute of Medical Sciences, India.	Women less than 63 days of gestation choosing medical abortion.	Misoprostol vaginal 400 μg 24 h after mifepristone (*N* = 100) vs. Misoprostol vaginal 400 μg 48 h after mifepristone (N = 100)
29.	Verma et al. 2017 [38]	Randomized controlled trial	Department of Obstetrics and Gynaecology, Hind Institute of Medical Sciences, India.	Women up to 63 days of gestation choosing medical abortion.	Misoprostol vaginal 400 μg simultaneously with mifepristone (N = 100) vs. Misoprostol vaginal 400 μg 48 h after mifepristone (N = 100)
30.	Von Hertzen et al. 2007 [39]	Randomized controlled trial	Eleven gynecological centers in six countries.	Women with single intra-uterine pregnancy less than or equal to 63 days verified by US, requesting termination of pregnancy.	1) Misoprostol 800 μg sublingual every 3 h × 3 doses (*N* = 517) vs. 2) Misoprostol 800 μg sublingual every 12 h × 3 doses (*N* = 516) vs. 3) Misoprostol 800 μg vaginal every 3 h × 3 doses (N = 516) vs. 4) Misoprostol 800 μg vaginal every 12 h × 3 doses (N = 517)
31.	Von Hertzen et al. 2009 [40]	Randomized controlled trial	Thirteen departments of obstetrics and gynecology in nine countries.	Women with 63 days or less gestation verified by ultrasound, requesting termination of pregnancy.	1) Mifepristone 100 mg + misoprostol 800 μg vaginal 24 h later (*N* = 545) vs. 2) Mifepristone 100 mg + misoprostol 800 μg vaginal 48 h later (*N* = 547) vs. 3) Mifepristone 200 mg + misoprostol 800 μg vaginal 24 h later (*N* = 544) vs. 4) Mifepristone 200 mg + misoprostol 800 μg vaginal 48 h later (N = 545)
32.	Von Hertzen et al. 2010 [10]	Randomized controlled trial	Fifteen obstetrics/gynecology departments in ten countries.	Women requesting legal termination of pregnancy at a gestation of up to 63 days.	1) Mifepristone 200 mg + misoprostol 400 μg sublingual 24 h later (*N* = 751) vs. 2) Mifepristone 200 mg + misoprostol 800 μg sublingual 24 h later (*N* = 752) vs. 3) Mifepristone 200 mg + misoprostol 400 μg vaginal 24 h later (N = 751) vs. 4) Mifepristone 200 mg + misoprostol 800 μg vaginal 24 h later (N = 751)
33.	Winikoff et al. 2008 [41]	Randomized controlled trial	Seven facilities in the United States.	Women seeking medical abortion with pregnancies not exceeding 63 days since the LMP on the day of the medical abortion. Gestational age was determined by LMP, clinical examination, and/or ultrasonography, as needed.	Misoprostol oral 800 μg 24–36 h after mifepristone (*N* = 482) vs. Misoprostol buccal800 μg 24–36 h after mifepristone (*N* = 484)

### Medical regimens

Different regimens of medical abortion management containing combination mifepristone misoprostol, or misoprostol alone were reviewed. Six studies compared combined mifepristone misoprostol vs. misoprostol alone, 6 studies compared different doses of misoprostol in combined regimens, 8 studies compared the timing interval between mifepristone and misoprostol in combined regimens, 13 compared routes of misoprostol in combined regimens, 2 compared various misoprostol alone regimens, and 1 study compared medical with suction evacuation.

1. ***Combination mifepristone misoprostol compared with misoprostol*alone**

Three studies compared combined with misoprostol alone regimens [6–8] (Table 2).

**Table 2:** Summary of findings of efficacy, safety and satisfaction of different regimens of medical abortion

Outcomes	Risk Ration (RR) Confidence Interval (CI)	Number of participants (Studies)	GRADE
***1. Combination mifepristone/misoprostol compared with misoprostol*alone**			
**A. Combination mifepristone-misoprostol compared with misoprostol alone (Table S1A)**			
Efficacy: ongoing pregnancy	**RR 0.16** (0.08 to 0.31)	922 (3 RCTs)	LOW
Efficacy: success	**RR 1.23** (1.16 to 1.30)	922 (2 RCTs)	VERY LOW
Safety	not estimable	(1 RCT)	VERY LOW
Satisfaction	**RR 1.13** (1.00 to 1.26)	820 (2 RCTs)	LOW
***2. Comparisons of different regimens of misoprostol when combined with mifepristone***			
***2.1. Comparison of misoprostol doses in combined regimen***			
**A. Misoprostol buccal 400 μg compared with 800** μg **in combined regimen (Table S2A)**			
Efficacy: ongoing pregnancy	**RR 0.16** (0.08 to 0.31)	1115 (1 RCT)	MODERATE
Efficacy: success	**RR 1.23** (1.16 to 1.30)	1115 (1 RCT)	MODERATE
Safety	**RR 1.00** (0.02 to 50.76)	1115 (1 RCT)	MODERATE
Satisfaction	**RR 0.99** (0.97 to 1.02)	1106 (1 RCT)	VERY LOW
**B. Misoprostol oral 400 μg twice compared with 400** μg **in combined regimen (Table S2B)**			
Efficacy: ongoing pregnancy	**RR 0.10** (0.01 to 0.80)	297 (1 RCT)	LOW
Efficacy: success	**RR 1.03** (0.86 to 1.23)	297 (1 RCT)	LOW
Safety	not estimable	(0 studies)	–
Satisfaction	**RR 1.03** (0.87 to 1.23)	293 (1 RCT)	LOW
**C. Misoprostol oral 800 μg single dose compared with 400** μg **twice in combined regimen (Table S2C)**			
Efficacy: ongoing pregnancy	**RR 0.88** (0.24 to 3.19)	637 (2 RCTs)	MODERATE
Efficacy: success	**RR 0.94** (0.89 to 0.99)	637 (2 RCTs)	MODERATE
Safety	not estimable	(0 studies)	–
Satisfaction	not estimable	(0 studies)	–
**D. Misoprostol sublingual 400 μg compared with 800** μg **in combined regimen (Table S2D)**			
Efficacy: ongoing pregnancy	**RR 3.44** (1.14 to 10.40)	1480 (1 RCT)	MODERATE
Efficacy: success	**RR 0.99** (0.92 to 1.07)	1480 (1 RCT)	MODERATE
Safety	not estimable	(0 studies)	–
Satisfaction	**RR 0.99** (0.92 to 1.07)	1475 (1 RCT)	LOW
**E. Misoprostol vaginal 400 μg compared with 800** μg **in combined regimen (Table S2E)**			
Efficacy: ongoing pregnancy	**RR 2.23** (0.98 to 5.11)	1482 (1 RCT)	MODERATE
Efficacy: success	**RR 0.97** (0.90 to 1.05)	1482 (1 RCT)	MODERATE
Safety	not estimable	(0 studies)	–
Satisfaction	**RR 0.99** (0.92 to 1.07)	1479 (1 RCT)	LOW
**F. Misoprostol oral 400 μg compared with 600** μg **in combined regimen (Table S2F)**			
Efficacy: ongoing pregnancy	**RR 0.33** (0.01 to 8.10)	638 (1 RCT)	LOW
Efficacy: success	**RR 1.01** (0.91 to 1.13)	638 (1 RCT)	LOW
Safety	**RR 0.33** (0.01 to 8.10)	638 (1 RCT)	LOW
Satisfaction	**RR 1.02** (0.91 to 1.16)	599 (1 RCT)	LOW
***2. 2. Comparison of dosing intervals between mifepristone and misoprostol in combined regimen***			
**A. Misoprostol 800 μg vaginal given < 8 h compared with > 24 h after mifepristone (Table S3A)**			
Efficacy: ongoing pregnancy	**RR 2.23** (0.69 to 7.20)	1525 (4 RCTs)	MODERATE
Efficacy: success	**RR 0.98** (0.91 to 1.06)	1525 (2 RCTs)	MODERATE
Safety	**RR 0.99** (0.02 to 49.60)	1100 (1 RCT)	MODERATE
Satisfaction	**RR 1.02** (0.87 to 1.18)	357 (1 RCT)	LOW
**B. Misoprostol 400–800 μg vaginal given 24 h compared with 48 h after mifepristone (Table S3B)**			
Efficacy: ongoing pregnancy	**RR 0.92** (0.40 to 2.12)	3301 (3 RCTs)	VERY LOW
Efficacy: success	**RR 0.99** (0.80 to 1.23)	192 (3 RCTs)	VERY LOW
Safety	not estimable	(0 studies)	–
Satisfaction	not estimable	(0 studies)	–
**C. Misoprostol 400 μg vaginal given concurrently compared with 24 h after mifepristone (Table S3C)**			
Efficacy: ongoing pregnancy	**RR 0.98** (0.02 to 49.25)	258 (2 RCTs)	VERY LOW
Efficacy: success	**RR 1.01** (0.84 to 1.21)	280 (2 RCTs)	VERY LOW
Safety	**RR 1.00** (0.02 to 50.01)	178 (2 RCTs)	VERY LOW
Satisfaction	**RR 1.02** (0.74 to 1.39)	80 (1 RCT)	VERY LOW
**D. Misoprostol 400 μg oral given < 8 h compared with 48 h after mifepristone (Table S3D)**			
Efficacy: ongoing pregnancy	**RR 8.34** (0.46 to 151.20)	100 (1 RCT)	VERY LOW
Efficacy: success	**RR 0.91** (0.66 to 1.25)	100 (1 RCT)	VERY LOW
Safety	**RR 1.96** (0.18 to 20.90)	100 (1 RCT)	VERY LOW
Satisfaction	not estimable	(0 studies)	–
***3. Comparisons of misoprostol routes in combined mifepristone-misoprostol regimen***			
**A. Misoprostol 400 μg sublingual compared with vaginal in combined regimen (Table S4A)**			
Efficacy: ongoing pregnancy	**RR 0.79** (0.39 to 1.55)	1479 (1 RCT)	MODERATE
Efficacy: success	**RR 1.01** (0.94 to 1.09)	1479 (1 RCT)	MODERATE
Safety	not estimable	(0 studies)	–
Satisfaction	**RR 1.00** (0.94 to 1.07)	1473 (1 RCT)	MODERATE
**B. Misoprostol 800 μg vaginal compared with sublingual in combined regimen (Table S4B)**			
Efficacy: ongoing pregnancy	**RR 0.50** (0.15 to 1.67)	1483 (1 RCT)	MODERATE
Efficacy: success	**RR 0.99** (0.92 to 1.07)	1483 (1 RCT)	MODERATE
Safety	not estimable	(0 studies)	–
Satisfaction	**RR 0.99** (0.92 to 1.07)	1481 (1 RCT)	VERY LOW
**C. Misoprostol 600/800 μg sublingual compared with 800** μg **vaginal in combined regimen (Table S4C)**			
Efficacy: ongoing pregnancy	**RR 0.15** (0.08 to 3.05)	346 (2 RCTs)	LOW
Efficacy: success	**RR 1.01** (0.87 to 1.18)	346 (2 RCTs)	LOW
Safety	**RR 1.00** (0.02 to 49.96)	224 (1 RCT)	LOW
Satisfaction	not estimable	(0 studies)	–
**D. Misoprostol 800 μg oral compared with vaginal in combined regimen (Table S4D)**			
Efficacy: ongoing pregnancy	**RR 6.70** (1.88 to 23.86)	1287 (3 RCTs)	MODERATE
Efficacy: success	**RR 0.94** (0.85 to 1.04)	1455 (3 RCTs)	MODERATE
Safety	**RR 0.35** (0.01 to 8.35)	263 (1 RCT)	VERY LOW
Satisfaction	not estimable	(0 studies)	–
**E. Misoprostol 400 μg oral compared with 800** μg **vaginal in combined regimen (Table S4E)**			
Efficacy: ongoing pregnancy	**RR 2.38** (0.34 to 16.81)	1378 (2 RCTs)	MODERATE
Efficacy: success	**RR 0.98** (0.91 to 1.04)	2025 (2 RCTs)	MODERATE
Safety	**RR 0.33** (0.01 to 8.15)	637 (1 RCT)	LOW
Satisfaction	**RR 1.02** (0.91 to 1.16)	599 (1 RCT)	LOW
**F. Misoprostol 800 μg buccal compared with sublingual in combined regimen (Table S4F)**			
Efficacy: ongoing pregnancy	**RR 0.98** (0.02 to 49.25)	90 (1 RCT)	VERY LOW
Efficacy: success	**RR 0.98** (0.73 to 1.33)	90 (1 RCT)	VERY LOW
Safety	**RR 0.98** (0.02 to 48.70)	178 (1 RCT)	VERY LOW
Satisfaction	not estimable	(0 studies)	–
**G. Misoprostol 400 μg buccal compared with sublingual in combined regimen (Table S4G)**			
Efficacy: ongoing pregnancy	**RR 1.55** (0.22 to 11.03)	539 (1 RCT)	LOW
Efficacy: success	**RR 0.98** (0.91 to 1.04)	539 (1 RCT)	LOW
Safety	**RR 0.33** (0.01 to 8.15)	539 (1 RCT)	LOW
Satisfaction	**RR 1.02** (0.91 to 1.16)	533 (1 RCT)	LOW
**H. Misoprostol 800 μg buccal compared with vaginal in combined regimen (Table S4H)**			
Efficacy: ongoing pregnancy	**RR 0.49** (0.09 to 2.68)	429 (1 RCT)	LOW
Efficacy: success	**RR 1.00** (0.87 to 1.15)	429 (1 RCT)	LOW
Safety	**RR 2.94** (0.12 to 71.80)	429 (1 RCT)	LOW
Satisfaction	**RR 0.98** (0.85 to 1.13)	423 (1 RCT)	LOW
**I. Misoprostol 800 μg oral compared with buccal in combined regimen (Table S4I)**			
Efficacy: ongoing pregnancy	**RR 3.61** (1.20 to 10.80)	847 (1 RCT)	LOW
Efficacy: success	**RR 0.97** (0.88 to 1.07)	847 (1 RCT)	LOW
Safety	**RR 0.33** (0.01 to 8.08)	847 (1 RCT)	LOW
Satisfaction	**RR 1.02** (0.91 to 1.12)	835 (1 RCT)	LOW
**J. Misoprostol 400 μg oral compared with sublingual in combined regimen (Table S4J)**			
Efficacy: ongoing pregnancy	**RR 0.44** (0.10 to 1.96)	564 (2 RCTs)	LOW
Efficacy: success	**RR 1.03** (0.99 to 1.07	564 (2 RCTs)	LOW
Safety	**RR 0.98** (0.01 to 49.14)	471 (1 RCT)	LOW
Satisfaction	**RR 1.02** (0.89 to 1.18)	470 (1 RCT)	LOW
***4. Comparisons of different misoprostol only regimens***			
**A. Misoprostol 400 μg oral every 3 h for 4 doses compared with 600** μg **vaginal misoprostol once (Table S5A)**			
Efficacy: ongoing pregnancy	**RR 1.50** (0.67 to 3.30)	76 (1 RCT)	VERY LOW
Efficacy: success	**RR 0.94** (0.52 to 1.70)	76 (1 RCT)	VERY LOW
Safety	not estimable	(0 studies)	–
Satisfaction	**RR 0.9** (0.5 to 1.6)	76 (1 RCT)	VERY LOW
**B. Misoprostol 800 μg oral every 6 h for 2 doses compared with 600 μg vaginal misoprostol once (Table S5B)**			
Efficacy: ongoing pregnancy	**RR 0.86** (0.28 to 2.59)	64 (1 RCT)	VERY LOW
Efficacy: success	**RR 1.12** (0.61 to 2.05)	64 (1 RCT)	VERY LOW
Safety	not estimable	(0 studies)	–
Satisfaction	**RR 1.01** (0.54 to 1.88)	64 (1 RCT)	VERY LOW
**C. Misoprostol 400 μg oral every 3 h for 4 doses compared with 800 μg oral misoprostol every 6 h for 2 doses (Table S5C)**			
Efficacy: ongoing pregnancy	**RR 1.75** (0.62 to 4.90)	60 (1 RCT)	VERY LOW
Efficacy: success	**RR 0.84** (0.44 to 1.59)	60 (1 RCT)	VERY LOW
Safety	not estimable	(0 studies)	–
Satisfaction	**RR 0.89** (0.46 to 1.72)	60 (1 RCT)	VERY LOW
**5. Comparison of medical and surgical management- Medical management with 800 μg vaginal misoprostol compared with surgical management (Table S6)**			
Efficacy: ongoing pregnancy	**RR 6.70** (1.88 to 23.86)	137 (1 RCT)	VERY LOW
Efficacy: success	**RR 1.02** (0.89 to 1.17)	137 (1 RCT)	VERY LOW
Safety	**RR 0.33** (0.01 to 8.04)	137 (1 RCT)	VERY LOW
Satisfaction	not estimable	(0 studies)	–

Women treated with a combined regimen had lower rates of ongoing pregnancy (RR 0.16 CI 95% 0.08–0.31, low certainty of evidence) and higher rates of successful abortion (RR 1.23 CI 95% 1.16–1.30, very low certainty of evidence) compared to women treated with a misoprostol only regimen. The combined regimen resulted in a higher rate of satisfaction compared with misoprostol only regimen (RR 1.13 CI 95% 1.00–1.26, low certainty of evidence) (Table S1, Additional file 3).

2. ***Comparisons of different regimens of misoprostol when combined with mifepristone***
  2.1. ***Comparison of misoprostol doses in combined regimen***

Six studies assessed different doses of misoprostol, using the same routes, in combined regimens. These included comparisons of 400 μg buccal vs. 800 μg buccal [9], 400 μg oral twice vs. 400 μg oral once [20], 800 μg oral once vs. 400 μg oral twice [22, 34], 400 μg sublingual vs. 800 μg sublingual [10], 400 μg vaginal vs. 800 μg vaginal [10] and 400 μg oral versus 600 μg oral [35] (Table 2).

Women treated with misoprostol 400 μg buccal had lower rates of ongoing pregnancy (RR 0.16 CI 95% 0.08–0.31, moderate certainty of evidence) and higher rates of successful abortion (RR 1.23 CI 95% 1.16–1.30, moderate certainty of evidence) compared to women taking 800 μg buccal [9].

For women taking a total of 800 μg oral misoprostol, there were lower rates of ongoing pregnancy (RR 0.10 CI 95% 0.01–0.80, low certainty of evidence) compared to women taking oral 400 μg [20]. Other studies that investigated 800 μg dosage of misoprostol showed comparable rates of successful abortion between 800 μg oral once and 400 μg oral twice (RR 0.94 CI 95% 0.89–0.99, moderate certainty of evidence) [22, 34].

Another significant finding was that women taking 400 μg sublingual misoprostol were more likely to experience ongoing pregnancy compared to the group who took 800 μg misoprostol (RR 3.44 CI 95% 1.14–10.40, moderate certainty of evidence) [10].

Although the remaining comparisons did not provide statistically significant findings, there was moderate certainty on the higher rates of ongoing pregnancy in the 400 μg vaginal misoprostol compared to the 800 μg vaginal misoprostol (Table 2). Safety and satisfaction appeared to be comparable throughout the groups (Table S2, Additional file 3).

2.2. ***Comparison of dosing intervals between mifepristone and misoprostol in combined regimen***

Eight studies assessed different time intervals between mifepristone and misoprostol dosing in the combined regimen. These include comparisons between < 8 h vs. > 24 h [11, 12], 24 h vs. 48 h [13, 32, 40], concurrent administration vs. 24 h [25, 38] and < 8 h vs. 48 h [37] (Table 2).

Administration of misoprostol within 8 h of mifepristone was found to have similar rates of successful abortion compared to 24-h (RR 0.98 CI 95% 0.91–1.06, moderate certainty of evidence) and 48-h intervals (RR 0.91 CI 95% 0.66–1.25, very low certainty of evidence) [11, 12, 37].

There may be little to no difference in rates of successful abortion between concurrent administration of misoprostol and a 24-h interval (RR 1.01 CI 95% 0.84–1.21, very low certainty of evidence) [25, 38]. There was no significant difference between 24-h and 48-h interval in terms of ongoing pregnancy and successful abortion [13, 32, 40]. All dosing interval comparisons showed similar safety and satisfaction rates (Table S3, Additional file 3).

3. ***Comparisons of misoprostol routes in combined mifepristone misoprostol regimen***

Thirteen studies assessed different routes of misoprostol in the combined regimen (Table 2).

Treatment with 800 μg oral misoprostol showed higher rates of ongoing pregnancy compared with vaginal (RR 6.70 CI 95% 1.88–23.86, moderate certainty of evidence) and buccal routes (RR 3.61 CI 95% 1.20–10.80, low certainty of evidence) [23, 33, 34, 41].

Women treated through sublingual route were found to have similar rates of successful abortion compared to those treated through vaginal route (RR 0.99 CI 95% 0.92–1.07, moderate certainty of evidence) [10].

There may be little to no difference in successful abortion rates among women treated through buccal route compared to those treated through sublingual (RR 0.98 CI 95% 0.73–1.33, very low certainty of evidence) or vaginal routes (RR 1.00 CI 95% 0.87–1.15, low certainty of evidence) [18, 28].

Safety and satisfaction rates of tested routes appears to be similar (Table S4, Additional file 3).

4. ***Comparisons of different misoprostol only regimens***

One study compared 7 different misoprostol only regimens [14] (Table 2). In this study, oral misoprostol 400 μg every 3 h administered for 4 doses was compared to vaginal misoprostol 600 μg once and oral misoprostol 800 μg administered every 6 h for 2 doses. In another arm, vaginal misoprostol 600 μg once was compared to oral misoprostol 800 μg administered every 6 h for 2 doses.

None of the study arms was more effective than the other. In addition, we were not able to compare the safety outcomes of these regimens (Table S5, Additional file 3).

5. ***Comparisons of medical*versus*surgical management***

One study compared surgical with medical management using a single dose of 800 μg vaginal misoprostol [29] (Table 2). Women treated with medical method showed higher rates of ongoing pregnancy than those receiving surgical management (RR 6.70 CI 95% 1.88–23.8). There was little to no difference in rates of successful abortion between the two methods (RR 1.02 CI 95% 0.89–1.17). There was a lower rate of serious adverse events and complications among women who received medical compared with those who received surgical management (RR 0.33 CI 95% 0.01–8.04). The certainty of evidence is very low for all reported outcomes (Table S6, Additional file 3).

## Discussion

In this review we identified 33 trials conducted across different settings with a total of 22,275 participants. We compared effectiveness, safety and acceptability of different combination and misoprostol only regimens. Acceptability was not explicitly reported; thus, we used satisfaction, which was reported in 25 of the included studies, as a proxy indicator.

The results of this review demonstrate that the majority of the studies compared different combination and misoprostol alone regimens in terms of dosing, route and frequency of administration. This reflects the fact that mifepristone has replaced older medications, such as methotrexate and gemeprost, when used in combination with misoprostol.

A combined regimen of mifepristone and misoprostol was found to be more effective in terms of lower rates of ongoing pregnancy and higher rates of successful abortion compared to the misoprostol alone regimen [6–8].

There have been multiple studies that focus on the combination regimen, comparing various misoprostol doses and routes and the interval between mifepristone and misoprostol.

When comparing different doses of misoprostol in the combined mifepristone misoprostol regimen, the included studies focused on the dosages of 400 μg and 800 μg. Comparing 400 μg to 800 μg buccal misoprostol [9], treatment with 400 μg misoprostol was found to be more effective (moderate certainty of evidence). On the other hand, administration of 800 μg oral misoprostol demonstrated more effectiveness than 400 μg oral misoprostol. Moreover, there is moderate certainty of evidence that 800 μg sublingual misoprostol is 3 times more effective than 400 μg [10]. Although there were multiple comparisons, it appears that the dosage of 800 μg of misoprostol in the combined mifepristone misoprostol regimen showed higher effectiveness with lower rates of ongoing pregnancy and higher rates of successful abortion. In addition, 800 μg were associated with higher rates of satisfaction [9, 10].

Review of studies that compared different dosing interval between mifepristone and misoprostol in combined regimen showed inconclusive results. Individual studies showed a 24-h interval to be more effective compared to either 8- or 48-h intervals [5, 9, 10, 32, 40]. However, we were not able to replicate these findings in the pooled analysis. We found similar rates of effectiveness between 24-h and 48-h intervals. In addition, the safety profile and satisfaction rates were not significantly different across intervals.

Comparing 8-h interval to 24-h and 48-h intervals showed that a shorter interval of misoprostol administration did not significantly compromise effectiveness [11, 12]. Furthermore, a 24-h interval was no more effective than concurrent administration. Our results align with existing evidence that demonstrates that concurrent administration can lead to higher satisfaction rates [5, 25, 38], while also impacting the number of visits required and time needed to complete the procedure [5]. Nonetheless, satisfaction rate was not consistently reported across studies. Thus, further research is needed to assess the impact of dosing interval on this outcome and how it relates to the acceptability of the procedure to women.

When comparing studies to determine optimal routes of misoprostol in combined mifepristone misoprostol regimen, there were mixed results. There is moderate certainty of evidence that oral misoprostol is significantly less effective than vaginal misoprostol [23, 33, 34]. Similarly, oral route was less effective than buccal route (low certainty of evidence) [41]. However, individual studies show that oral administration of misoprostol in the combined regimen leads to better overall satisfaction [18, 23, 33, 34].

Buccal route was as effective as sublingual and vaginal route and there was no significant difference between sublingual and vaginal routes [18, 28, 31]. Given the findings of the non-significant differences between the routes, women should be given the full range options factoring in their satisfaction towards a particular treatment regimen.

A review of one study with 7 different arms comparing misoprostol only regimens failed to demonstrate superiority of one regimen over the others. This potentially means that the compared regimens are equally effective and at this time no conclusions can be made without additional studies evaluating misoprostol only regimens. This is important in order to address the needs of those who cannot afford or access mifepristone [14].

Compared to surgical method, medical management had significantly higher rates of ongoing pregnancy. Lower rates of serious adverse events and complications were observed with medical compared to surgical methods [29]. However, interpretation of this finding requires caution as it was based on only one trial and certainty of evidence was very low.

One study comparing oral versus vaginal misoprostol reported one woman in the vaginal arm who died from a systemic *Clostridium sordellii* infection [35]. However, in general, the rates of serious adverse events reported in our review are very low, thus we cannot draw definitive conclusions related to adverse events.

### Strengths and limitations

This review has several strengths. We used a comprehensive and replicable search strategy to identify relevant articles. In addition, the included studies were conducted across different settings. We employed the GRADE system that can assist health care providers, program managers and policy makers to design and implement best practice recommendations and guidelines.

Limitations of this review include the inclusion of only RCTs and using satisfaction as a proxy for acceptability. Specifically, inclusion of observational studies could be more informative about client satisfaction and acceptability of treatment regimens. We were not able to demonstrate statistically significant differences for various dosing intervals and routes of misoprostol administration in combination or in misoprostol alone regimens. There are only a limited number of studies for some of the comparisons (medical vs. surgical, misoprostol only regimens). In addition, some of the included studies have a high risk of performance and detection bias. Thus, we recommend future research studies to consider blinding of outcome assessor as it is feasible to blind the individual who is assessing the success of the abortion (whether by history, physical exam or ultrasound) and this in turn can improve the quality of data.

## Conclusion

In this systematic review, we establish that medical methods of abortion are effective, safe and acceptable for termination of pregnancy of ≤63 days of gestation. The combined regimen of mifepristone and misoprostol was more effective than the misoprostol alone regimen. In the combined regimen, the dosage of 800 μg misoprostol was more effective than 400 μg. Although there were no significant differences in the dosing interval and the routes of misoprostol, the additive information on the certainty of evidence and consideration of women’s satisfaction, suggest that a 24-h interval and offering different routes of administration are effective, safe and acceptable options for medical abortion. This further highlights the fact that in many cases, demonstrating that one option does not lead to statistically significant better outcomes than the other allows for making clinical decisions based on an individual’s preference*.* More robust studies evaluating both the different combination and misoprostol alone regimens are needed to strengthen existing evidence as well as assess patient perspectives towards a particular regimen.

## Supplementary information

**Additional file 1.** Search strategies from electronic databases.

**Additional file 2.** Risk of bias.

**Additional file 3 Table S1.** Regimens for medical abortion ≤63 days. **Table S2.** Comparison of misoprostol doses in combined regimen. **Table S3.** Comparison of dosing intervals between mifepristone and misoprostol in combined regimen. **Table S4.** Comparison of misoprostol routes in combined mifepristone-misoprostol regimen. **Table S5.** Comparison of different misoprostol only regimens. **Table S6.** Comparison of medical and surgical management- Medical management with 800 μg vaginal misoprostol compared with surgical management.

## Data Availability

The datasets supporting the conclusions of this article are included within the article and its additional files.

## Abbreviations

CI: Confidence Interval
GRADE: Grading of Recommendations, Assessment, Development and Evaluations
LMP: Last Menstrual Period
MeSH: Medical Subject Headings
PRISMA: Preferred Reporting Items for Systematic Reviews and Meta-Analyses
RCTs: Randomized Controlled Trials
RR: Risk Ratio
WHO: World Health Organization
