# Supplementary material for: Medical termination for pregnancy in early first trimester (≤ 63 days) using combination of mifepristone and misoprostol or misoprostol alone: a systematic review
Source: BMC Womens Health. 2020 Jul 7;20:142. doi: 10.1186/s12905-020-01003-8 (PMC7339463; doi:10.1186/s12905-020-01003-8)
Supplement: Supplementary file 1 — Additional file 1. Search strategies from electronic databases. [file 12905_2020_1003_MOESM1_ESM.docx]

**Search strategies from electronic databases**

**PubMed**

| **S. No.** | **Concept** | **Search terms** |
| --- | --- | --- |
| 1 | Induced abortion | "abortion, induced"[MeSH Terms] OR “Abortion, Criminal"[Mesh] OR "Abortion, Septic"[Mesh] OR "Abortion Applicants"[Mesh] OR abortion*[Text Word] OR ("pregnan*"[TW] AND terminat*[TW]) OR (Abort*[TW] AND Pregnan*[TW]) |
| 2 | Gestational age | Gestational age[MeSH] OR Pregnancy[MeSH] OR Pregnancy Trimester, First[MeSH] OR Pregnancy trimesters[MeSH] OR first trimester[tiab] OR Gestation*[tiab] OR Last menstrual period[tiab] OR "70 days"[tiab] |
| 3 | Medical methods for abortion (i.e., misoprostol, mifepristone) | Misoprostol[MeSH] OR Misoprostol[tiab] OR mifepristone[tiab] OR Mifepristone[MeSH] OR RU-486 OR RU486 OR R38486 [TIAB] OR RU 38486 [TIAB] OR RU-38486 [TIAB] OR Mifegyne [TIAB] OR Mifégyne [TIAB] OR Glefos [TIAB] OR Mifeprex [TIAB] OR Cytotec [TIAB] OR ZK 98296 [TIAB] OR ZK98296 [TIAB] OR ZK-98296 [TIAB] OR SC 29333 [TIAB] OR SC 30249 [TIAB] OR SC29333 [TIAB] OR SC-29333 [TIAB] OR SC30249 [TIAB] OR SC-30249 [TIAB]OR antiprogesterone[TIAB] OR antiprogesterone[TIAB] OR abortifacient[TIAB] OR "abortifacient agent”[TIAB] OR "abortifacient agents” [TIAB] |
| 4 | Surgical methods of abortion | (Dilatation and curettage[MeSH] OR Vacuum Curettage[MeSH] OR Surgical abortion[tiab] OR Manual vacuum aspiration OR (vacuum[tiab] AND aspiration[tiab]) OR Curettage[tiab] OR Surgical termination of pregnancy[tiab] OR Dilatation and evacuation[tiab] OR Dilation and evacuation[tiab] OR Suction aspiration[tiab] OR Aspiration abortion [tiab] OR Suction curettage[tiab] OR Vacuum curettage[tiab]) |
| 5 | Randomized clinical trials | ((randomized controlled trial[pt]) OR (controlled clinical trial[pt]) OR (randomised[tiab] OR randomized[tiab]) OR (placebo[tiab]) OR (drug therapy[sh]) OR (randomly[tiab]) OR (trial[tiab]) OR (groups[tiab])) NOT (animals[mh] NOT humans[mh]) |

**EMBASE**

| **S. No.** | **Concept** | **Search terms** |
| --- | --- | --- |
| 1 | Induced abortion | ('induced abortion'/de OR 'legal abortion'/de OR 'illegal abortion'/de OR 'therapeutic abortion'/de OR 'septic abortion'/de OR 'abortion'/exp OR 'abortion'/de OR 'pregnancy termination'/de OR (pregnant* AND terminat*) OR (abort* AND pregnant*) OR (abort* AND pregnanc*) OR (terminat* AND pregnanc*)) |
| 2 | Medical methods for abortion (i.e., misoprostol, mifepristone) | ('mifepristone'/de OR 'mifepristone':ab,ti OR 'misoprostol'/de OR 'misoprostol':ab,ti OR 'methotrexate'/de or 'methotrexate':ab,ti OR ‘prostaglandin’/de or ‘prostaglandin’:ab,ti OR ‘prostaglandin E2’/de OR 'prostaglandin f2 alpha'/de OR 'carboprostacyclin'/de OR ‘sulprostone’/de OR ‘gemeprost’/de OR ‘metenoprost’/de OR ‘lilopristone’/de OR ‘onapristone’/de OR ‘epostane’/de OR ‘oxytocin’/de OR ‘sodium chloride’/de OR ‘urea’:ab,ti OR ‘iodine’:ab,ti OR 'ethacridine lactate'/de OR ‘ethacridine’/de OR 'antigestagen':ab,ti OR ‘abortive agent’/de) |
| 3 | Randomized clinical trials | ('crossover procedure':de OR 'double-blind procedure':de OR 'randomized controlled trial':de OR  'single-blind procedure':de OR (random* OR  factorial* OR crossover* OR cross NEXT/1 over* OR placebo* OR doubl* NEAR/1 blind* OR singl* NEAR/1 blind* OR assign* OR allocat* OR volunteer*):de,ab,ti) |
