# Supplementary material for: Medical termination for pregnancy in early first trimester (≤ 63 days) using combination of mifepristone and misoprostol or misoprostol alone: a systematic review
Source: BMC Womens Health. 2020 Jul 7;20:142. doi: 10.1186/s12905-020-01003-8 (PMC7339463; doi:10.1186/s12905-020-01003-8)
Supplement: Supplementary file 2 — Additional file 2. Risk of bias. [file 12905_2020_1003_MOESM2_ESM.docx]

**Risk of bias**

| **Study** | **Random sequence generation (selection bias)** | **Allocation concealment (selection bias)** | **Blinding of participants & personnel (performance bias)** | **Blinding of outcome assessments (detection bias)** | **Incomplete outcome data (attrition bias)** | **Selective reporting (reporting bias)** | **Other bias** |
| --- | --- | --- | --- | --- | --- | --- | --- |
| Blanchard et al 2005 | **+** | **+** | **-** | **-** | **+** | **+** | **+** |
| Blum et al 2012 | **+** | **+** | **+** | **?** | **+** | **+** | **?** |
| Chai et al 2013 | **+** | **+** | **+** | **+** | **+** | **+** | **+** |
| Chawdhary et al 2009 | **-** | **-** | **-** | **-** | **+** | **+** | **+** |
| Chong et al 2012 | **+** | **+** | **+** | **?** | **+** | **+** | **?** |
| Coyaji et al 2007 | **+** | **+** | **?** | **?** | **+** | **+** | **+** |
| Creinin et al 2007 | **+** | **+** | **-** | **-** | **+** | **+** | **-** |
| Dahiya et al 2011 | **+** | **+** | **-** | **-** | **+** | **+** | **-** |
| Dahiya et al 2012 | **?** | **?** | **-** | **-** | **+** | **+** | **+** |
| el-Refaey et al 1994 | **+** | **+** | **-** | **-** | **-** | **+** | **+** |
| El-Refaey et al 1995 | **+** | **+** | **-** | **-** | **-** | **+** | **-** |
| Fekih et al 2010 | **+** | **+** | **-** | **-** | **+** | **+** | **?** |
| Goel et al 2011 | **+** | **+** | **-** | **-** | **+** | **+** | **?** |
| Guest et al 2007 | **+** | **+** | **-** | **-** | **-** | **+** | **-** |
| Hamoda et al 2005 | **+** | **+** | **-** | **-** | **+** | **+** | **+** |
| Jain et al 2002 | **+** | **+** | **+** | **?** | **+** | **+** | **+** |
| Middleton et al 2005 | **+** | **+** | **-** | **-** | **+** | **+** | **-** |
| Ngoc et al 2011 | **+** | **-** | **+** | **?** | **+** | **-** | **?** |
| Prasad et al 2009 | **-** | **-** | **-** | **-** | **+** | **-** | **?** |
| Raghavan et al 2009 | **+** | **+** | **-** | **-** | **+** | **+** | **+** |
| Raghavan et al 2010 | **+** | **?** | **-** | **-** | **+** | **+** | **-** |
| Schaff et al 2000 | **+** | **+** | **-** | **?** | **+** | **+** | **+** |
| Schaff et al 2001 | **+** | **+** | **-** | **-** | **+** | **+** | **-** |
| Schaff et al 2002 | **+** | **?** | **-** | **-** | **+** | **-** | **-** |
| Shannon et al 2006 | **+** | **?** | **-** | **-** | **+** | **+** | **+** |
| Tang et al 2003 | **+** | **?** | **+** | **+** | **+** | **+** | **-** |
| Tendler et al 2015 | **+** | **?** | **-** | **?** | **+** | **+** | **-** |
| Verma et al 2011 | **+** | **?** | **-** | **-** | **+** | **+** | **+** |
| Verma et al 2017 | **-** | **-** | **-** | **-** | **+** | **-** | **+** |
| von Hertzen et al 2007 | **+** | **+** | **-** | **-** | **+** | **+** | **?** |
| von Hertzen et al 2009 | **+** | **+** | **+** | **?** | **+** | **+** | **?** |
| von Hertzen et al 2010 | **+** | **+** | **+** | **?** | **+** | **+** | **-** |
| Winikoff et al 2008 | **+** | **+** | **-** | **?** | **+** | **+** | **+** |
