# Supplementary material for: Medical termination for pregnancy in early first trimester (≤ 63 days) using combination of mifepristone and misoprostol or misoprostol alone: a systematic review
Source: BMC Womens Health. 2020 Jul 7;20:142. doi: 10.1186/s12905-020-01003-8 (PMC7339463; doi:10.1186/s12905-020-01003-8)
Supplement: Supplementary file 3 — Additional file 3 Table S1. Regimens for medical abortion ≤63 days. Table S2. Comparison of misoprostol doses in combined regimen. Table S3. Comparison of dosing intervals between mifepristone and misoprostol in combined regimen. Table S4. Comparison of misoprostol routes in combined mifepristone-misoprostol regimen. Table S5. Comparison of different misoprostol only regimens. Table S6. Comparison of medical and surgical management- Medical management with 800 μg vaginal misoprostol compared with surgical management. [file 12905_2020_1003_MOESM3_ESM.docx]

**Table S1. Regimens for medical abortion ≤ 63 days**

1. **Combination mifepristone-misoprostol compared with misoprostol alone**

| **Outcomes** | **Anticipated absolute effects^*^ (95% CI)** | | **Relative effect (95% CI)** | **№ of participants  (studies)** | **GRADE** | **Comments** |
| --- | --- | --- | --- | --- | --- | --- |
|  | **Risk with misoprostol alone** | **Risk with combined mifepristone & misoprostol** |  |  |  |  |
| Efficacy: ongoing pregnancy (Efficacy) | 139 per 1,000 | **22 per 1,000** (11 to 43) | **RR 0.16** (0.08 to 0.31) | 922 (3 RCTs) ^1,2,3^ | ⨁⨁◯◯ LOW | Our confidence in the direct estimate is limited. The true effect may be substantially different from the estimate of the effect. |
| Efficacy: completed without surgical intervention | 768 per 1,000 | **945 per 1,000** (891 to 998) | **RR 1.23** (1.16 to 1.30) | 922 (2 RCTs) ^1,2^ | ⨁◯◯◯ VERY LOW | We are uncertain about the effect on this outcome because the certainty of the evidence is very low. |
| Safety: serious adverse events and complications | 0 per 1,000 | **0 per 1,000** (0 to 0) | not estimable | (1 RCT) ^1^ | ⨁◯◯◯ VERY LOW | We are uncertain about the effect on this outcome because the certainty of the evidence is very low. |
| Expulsion time | 0 per 1,000 | **0 per 1,000** (0 to 0) | not estimable | (0 studies) | - | No direct evidence identified. |
| Side effects: bleeding | 286 per 1,000 | **411 per 1,000** (337 to 497) | **RR 1.44** (1.18 to 1.74) | 805 (2 RCTs) ^1,2^ | ⨁⨁◯◯ LOW | Our confidence in the direct estimate is limited. The true effect may be substantially different from the estimate of the effect. |
| Side effects: pain | 322 per 1,000 | **312 per 1,000** (254 to 383) | **RR 0.97** (0.79 to 1.19) | 805 (2 RCTs) ^1,2^ | ⨁⨁◯◯ LOW | Our confidence in the direct estimate is limited. The true effect may be substantially different from the estimate of the effect. |
| Side effects: vomiting | 229 per 1,000 | **220 per 1,000** (174 to 277) | **RR 0.96** (0.76 to 1.21) | 820 (2 RCTs) ^1,2^ | ⨁⨁⨁◯ MODERATE | Use of combined mifepristone and misoprostol probably slightly reduces emesis. |
| Satisfaction | 747 per 1,000 | **844 per 1,000** (747 to 941) | **RR 1.13** (1.00 to 1.26) | 820 (2 RCTs) ^1,2^ | ⨁⨁◯◯ LOW | Our confidence in the direct estimate is limited. The true effect may be substantially different from the estimate of the effect. |
| ***The risk in the intervention group** (and its 95% confidence interval) is based on the assumed risk in the comparison group and the **relative effect** of the intervention (and its 95% CI).   **CI:** Confidence interval; **RR:** Risk ratio. **Studies:** Blum 2012^1^, Ngoc 2011^2^ and Dahiya 2012^3^ | | | | | | |

1. **Mifepristone and vaginal misoprostol in combination compared with 800 μg vaginal misoprostol alone**

| **Outcomes** | **Anticipated absolute effects^*^ (95% CI)** | | **Relative effect (95% CI)** | **№ of participants  (studies)** | **GRADE** | **Comments** |
| --- | --- | --- | --- | --- | --- | --- |
|  | **Risk with 800 μg vaginal misoprostol alone** | **Risk with combined mifepristone and misoprostol** |  |  |  |  |
| Efficacy: ongoing pregnancy | 51 per 1,000 | **5 per 1,000** (1 to 41) | **RR 0.10** (0.01 to 0.80) | 344 (2 RCTs) ^1,2^ | ⨁◯◯◯ VERY LOW | We are uncertain about the effect on this outcome because the certainty of the evidence is very low. |
| Efficacy: completed without surgical intervention | 860 per 1,000 | **903 per 1,000** (671 to 1,000) | **RR 1.05** (0.78 to 1.41) | 100 (2 RCT) ^1,2^ | ⨁◯◯◯ VERY LOW | We are uncertain about the effect on this outcome because the certainty of the evidence is very low. |
| Safety: serious adverse events and complications | 0 per 1,000 | **0 per 1,000** (0 to 0) | **RR 1.05** (0.02 to 52.49) | 244 (1 RCT) ^2^ | ⨁◯◯◯ VERY LOW | We are uncertain about the effect on this outcome because the certainty of the evidence is very low. |
| Expulsion time | 0 per 1,000 | **0 per 1,000** (0 to 0) | not estimable | (0 studies) | - | No direct evidence identified |
| Side effects: bleeding | 220 per 1,000 | **24 per 1,000** (2 to 178) | **RR 0.11** (0.01 to 0.81) | 100 (1 RCT) ^1^ | ⨁◯◯◯ VERY LOW | We are uncertain about the effect on this outcome because the certainty of the evidence is very low. |
| Side effects: pain | 171 per 1,000 | **171 per 1,000** (106 to 274) | **RR 1.00** (0.62 to 1.60) | 344 (2 RCTs) ^1,2^ | ⨁◯◯◯ VERY LOW | We are uncertain about the effect on this outcome because the certainty of the evidence is very low. |
| Side effects: vomiting | 211 per 1,000 | **326 per 1,000** (226 to 465) | **RR 1.54** (1.07 to 2.20) | 344 (2 RCTs) ^1,2^ | ⨁◯◯◯ VERY LOW | We are uncertain about the effect on this outcome because the certainty of the evidence is very low. |
| Satisfaction | 0 per 1,000 | **0 per 1,000** (0 to 0) | not estimable | (0 studies) | - | No direct evidence identified |
| ***The risk in the intervention group** (and its 95% confidence interval) is based on the assumed risk in the comparison group and the **relative effect** of the intervention (and its 95% CI).   **CI:** Confidence interval; **RR:** Risk ratio. **Studies:** Chawdhary 2009^1^ and Jain 2002^2^ | | | | | | |

1. **Mifepristone and 400 μg oral misoprostol in combination compared with 800 μg sublingual misoprostol alone every 4 hours**

| **EARLYMA1c** | | | | | | |
| --- | --- | --- | --- | --- | --- | --- |
| **Outcomes** | **Anticipated absolute effects^*^ (95% CI)** | | **Relative effect (95% CI)** | **№ of participants  (studies)** | **GRADE** | **Comments** |
|  | **Risk with 800 μg sublingual misoprostol alone every 4 hours** | **Risk with combined mifepristone and 400 μg oral misoprostol** |  |  |  |  |
| Efficacy: ongoing pregnancy | 8 per 1,000 | **8 per 1,000** (0 to 125) | **RR 1.00** (0.06 to 15.81) | 252 (1 RCT) ^1^ | ⨁◯◯◯ VERY LOW | We are uncertain about the effect on this outcome because the certainty of the evidence is very low. |
| Efficacy: completed without surgical intervention | 921 per 1,000 | **930 per 1,000** (773 to 1,000) | **RR 1.01** (0.84 to 1.21) | 252 (1 RCT) ^1^ | ⨁◯◯◯ VERY LOW | We are uncertain about the effect on this outcome because the certainty of the evidence is very low. |
| Safety: serious adverse events and complications | 0 per 1,000 | **0 per 1,000** (0 to 0) | **RR 1.00** (0.02 to 50.01) | 252 (1 RCT) ^1^ | ⨁◯◯◯ VERY LOW | We are uncertain about the effect on this outcome because the certainty of the evidence is very low. |
| Expulsion time | 0 per 1,000 | **0 per 1,000** (0 to 0) | not estimable | (0 studies) | - | No direct evidence identified. |
| Side effects: bleeding | 63 per 1,000 | **99 per 1,000** (43 to 232) | **RR 1.56** (0.67 to 3.65) | 252 (1 RCT) ^1^ | ⨁◯◯◯ VERY LOW | We are uncertain about the effect on this outcome because the certainty of the evidence is very low. |
| Side effects: pain | 373 per 1,000 | **269 per 1,000** (179 to 403) | **RR 0.72** (0.48 to 1.08) | 252 (1 RCT) ^1^ | ⨁⨁◯◯ LOW | Our confidence in the direct estimate is limited. The true effect may be substantially different from the estimate of the effect. |
| Side effects: vomiting | 0 per 1,000 | **0 per 1,000** (0 to 0) | not estimable | ( studies) | - | No direct evidence identified. |
| Satisfaction | 921 per 1,000 | **939 per 1,000** (783 to 1,000) | **RR 1.02** (0.85 to 1.22) | 252 (1 RCT) ^1^ | ⨁◯◯◯ VERY LOW | We are uncertain about the effect on this outcome because the certainty of the evidence is very low. |
| ***The risk in the intervention group** (and its 95% confidence interval) is based on the assumed risk in the comparison group and the **relative effect** of the intervention (and its 95% CI).   **CI:** Confidence interval; **RR:** Risk ratio. **Studies:** Fekih 2010^1^ | | | | | | |

**Table S2. Comparison of misoprostol doses in combined regimen**

1. **Misoprostol buccal 400 μg compared with 800 μg in combined regimen**

| **Outcomes** | **Anticipated absolute effects^*^ (95% CI)** | | **Relative effect (95% CI)** | **№ of participants  (studies)** | **GRADE** | **Comments** |
| --- | --- | --- | --- | --- | --- | --- |
|  | **Risk with 800 μg buccal misoprostol in combined regimen** | **Risk with 400 μg buccal misoprostol in combined regimen** |  |  |  |  |
| Efficacy: ongoing pregnancy (Efficacy) | 9 per 1,000 | **1 per 1,000** (1 to 3) | **RR 0.16** (0.08 to 0.31) | 1115 (1 RCT) ^1^ | ⨁⨁⨁◯ MODERATE | Use of 400 μg misoprostol buccally probably slightly reduces the risk of ongoing pregnancy. |
| Efficacy: completed without surgical intervention | 964 per 1,000 | **1000 per 1,000** (1,000 to 1,000) | **RR 1.23** (1.16 to 1.30) | 1115 (1 RCT) ^1^ | ⨁⨁⨁◯ MODERATE | Use of 400 μg misoprostol buccally probably slightly reduces the risk of being completed without surgical intervention. |
| Safety: serious adverse events and complications | 0 per 1,000 | **0 per 1,000** (0 to 0) | **RR 1.00** (0.02 to 50.76) | 1115 (1 RCT) ^1^ | ⨁⨁⨁◯ MODERATE | Use of 800 μg misoprostol buccally probably does not alter the risk of SAE |
| Expulsion time | 0 per 1,000 | **0 per 1,000** (0 to 0) | not estimable | (0 studies) | - |  |
| Side effects: bleeding | 11 per 1,000 | **15 per 1,000** (13 to 19) | **RR 1.44** (1.18 to 1.74) | 1115 (1 RCT) ^1^ | ⨁⨁◯◯ LOW | Our confidence in the direct estimate is limited. The true effect may be substantially different from the estimate of the effect. |
| Side effects: pain | 809 per 1,000 | **777 per 1,000** (728 to 825) | **RR 0.96** (0.90 to 1.02) | 1115 (1 RCT) ^1^ | ⨁⨁◯◯ LOW | Our confidence in the direct estimate is limited. The true effect may be substantially different from the estimate of the effect. |
| Side effects: vomiting | 220 per 1,000 | **158 per 1,000** (123 to 202) | **RR 0.72** (0.56 to 0.92) | 1115 (1 RCT) ^1^ | ⨁⨁◯◯ LOW | Our confidence in the direct estimate is limited. The true effect may be substantially different from the estimate of the effect. |
| Satisfaction | 962 per 1,000 | **953 per 1,000** (933 to 981) | **RR 0.99** (0.97 to 1.02) | 1106 (1 RCTs) ^1^ | ⨁◯◯◯ VERY LOW | We are uncertain about the effect on this outcome because the certainty of the evidence is very low. |
| ***The risk in the intervention group** (and its 95% confidence interval) is based on the assumed risk in the comparison group and the **relative effect** of the intervention (and its 95% CI).   **CI:** Confidence interval; **RR:** Risk ratio. **Studies:** Chong 2012^1^ | | | | | | |

1. **Misoprostol oral 400 μg twice compared with 400 μg in combined regimen**

| **Outcomes** | **Anticipated absolute effects^*^ (95% CI)** | | **Relative effect (95% CI)** | **№ of participants  (studies)** | **GRADE** | **Comments** |
| --- | --- | --- | --- | --- | --- | --- |
|  | **Risk 400 μg oral misoprostol once in combined regimen** | **Risk with 400 μg oral misoprostol twice in combined regimen** |  |  |  |  |
| Efficacy: ongoing pregnancy | 68 per 1,000 | **7 per 1,000** (1 to 54) | **RR 0.10** (0.01 to 0.80) | 297 (1 RCT) ^1^ | ⨁⨁◯◯ LOW | Our confidence in the direct estimate is limited. The true effect may be substantially different from the estimate of the effect. |
| Efficacy: completed without surgical intervention | 864 per 1,000 | **890 per 1,000** (743 to 1,000) | **RR 1.03** (0.86 to 1.23) | 297 (1 RCT) ^1^ | ⨁⨁◯◯ LOW | Our confidence in the direct estimate is limited. The true effect may be substantially different from the estimate of the effect. |
| Safety: serious adverse events and complications | 0 per 1,000 | **0 per 1,000** (0 to 0) | not estimable | (0 studies) | - | No direct evidence identified |
| Expulsion time | 0 per 1,000 | **0 per 1,000** (0 to 0) | not estimable | (1 RCT) ^1^ | - | Expulsion time for the intervention group was 179.21 min as compared with 193.91 min for the single dose group. |
| Side effects: bleeding | 553 per 1,000 | **548 per 1,000** (426 to 703) | **RR 0.99** (0.77 to 1.27) | 300 (1 RCT) ^1^ | ⨁⨁◯◯ LOW | Our confidence in the direct estimate is limited. The true effect may be substantially different from the estimate of the effect. |
| Side effects: pain | 873 per 1,000 | **882 per 1,000** (734 to 1,000) | **RR 1.01** (0.84 to 1.20) | 300 (1 RCT) ^1^ | ⨁⨁◯◯ LOW | Our confidence in the direct estimate is limited. The true effect may be substantially different from the estimate of the effect. |
| Side effects: vomiting | 0 per 1,000 | **0 per 1,000** (0 to 0) | **RR 1.00** (0.02 to 50.00) | 300 (1 RCT) ^1^ | ⨁⨁◯◯ LOW | Our confidence in the direct estimate is limited. The true effect may be substantially different from the estimate of the effect. |
| Satisfaction | 882 per 1,000 | **908 per 1,000** (767 to 1,000) | **RR 1.03** (0.87 to 1.23) | 293 (1 RCT) ^1^ | ⨁⨁◯◯ LOW | Our confidence in the direct estimate is limited. The true effect may be substantially different from the estimate of the effect. |
| ***The risk in the intervention group** (and its 95% confidence interval) is based on the assumed risk in the comparison group and the **relative effect** of the intervention (and its 95% CI).   **CI:** Confidence interval; **RR:** Risk ratio. **Studies:** Coyji 2007^1^ | | | | | | |

1. **Misoprostol oral 800 μg single dose compared with 400 μg twice in combined regimen**

| **Outcomes** | **Anticipated absolute effects^*^ (95% CI)** | | **Relative effect (95% CI)** | **№ of participants  (studies)** | **GRADE** | **Comments** |
| --- | --- | --- | --- | --- | --- | --- |
|  | **Risk with 400 μg oral misoprostol twice in combined regimen** | **Risk with 800 μg single dose oral misoprostol in combined regimen** |  |  |  |  |
| Efficacy: ongoing pregnancy | 15 per 1,000 | **13 per 1,000** (3 to 47) | **RR 0.88** (0.24 to 3.19) | 637 (2 RCTs) ^1,2^ | ⨁⨁⨁◯ MODERATE | There is probably no difference in this outcome. |
| Efficacy: completed without surgical intervention | 918 per 1,000 | **863 per 1,000** (817 to 909) | **RR 0.94** (0.89 to 0.99) | 637 (2 RCTs) ^1,2^ | ⨁⨁⨁◯ MODERATE | There is probably a slightly reduced risk of the procedure being completed without surgical intervention when miso 400 po is used twice. |
| Safety: serious adverse events and complications | 0 per 1,000 | **0 per 1,000** (0 to 0) | not estimable | (0 studies) | - | No direct evidence identified. |
| Expulsion time | 0 per 1,000 | **0 per 1,000** (0 to 0) | not estimable | (0 studies) | - | No direct evidence identified. |
| Side effects: bleeding | 40 per 1,000 | **27 per 1,000** (4 to 157) | **RR 0.67** (0.11 to 3.93) | 150 (1 RCT) ^1^ | ⨁◯◯◯ VERY LOW | We are uncertain about the effect on this outcome because the certainty of the evidence is very low. |
| Side effects: pain | 387 per 1,000 | **363 per 1,000** (232 to 572) | **RR 0.94** (0.60 to 1.48) | 150 (1 RCT) ^1^ | ⨁◯◯◯ VERY LOW | We are uncertain about the effect on this outcome because the certainty of the evidence is very low. |
| Side effects: vomiting | 307 per 1,000 | **371 per 1,000** (233 to 595) | **RR 1.21** (0.76 to 1.94) | 150 ( RCTs) ^1^ | ⨁◯◯◯ VERY LOW | We are uncertain about the effect on this outcome because the certainty of the evidence is very low. |
| Satisfaction | 0 per 1,000 | **0 per 1,000** (0 to 0) | not estimable | (0 studies) | - | No direct evidence identified. |
| ***The risk in the intervention group** (and its 95% confidence interval) is based on the assumed risk in the comparison group and the **relative effect** of the intervention (and its 95% CI).   **CI:** Confidence interval; **RR:** Risk ratio. **Studies:** el-Refaey 1994^1^ and Schaff 2002^2^ | | | | | | |

1. **Misoprostol sublingual 400 μg compared with 800 μg in combined regimen**

| **Outcomes** | **Anticipated absolute effects^*^ (95% CI)** | | **Relative effect (95% CI)** | **№ of participants  (studies)** | **GRADE** | **Comments** |
| --- | --- | --- | --- | --- | --- | --- |
|  | **Risk with 800 μg sublingual misoprostol in combined regimen** | **Risk with 400 μg sublingual misoprostol in combined regimen** |  |  |  |  |
| Efficacy: ongoing pregnancy | 5 per 1,000 | **19 per 1,000** (6 to 56) | **RR 3.44** (1.14 to 10.40) | 1480 (1 RCT) ^1^ | ⨁⨁⨁◯ MODERATE | There is probably a slightly increased risk of ongoing pregnancy when 400 μg versu 800 μg misoprostol is used sublingually. |
| Efficacy: completed without surgical intervention | 939 per 1,000 | **930 per 1,000** (864 to 1,000) | **RR 0.99** (0.92 to 1.07) | 1480 (1 RCT) ^1^ | ⨁⨁⨁◯ MODERATE | There is probably no difference in the outcome when a dose of 400 μg or 800 μg of misoprostol is used sublingually. |
| Safety: serious adverse events and complications | 0 per 1,000 | **0 per 1,000** (0 to 0) | not estimable | (0 studies) | - | No direct evidence identified. |
| Expulsion time | 0 per 1,000 | **0 per 1,000** (0 to 0) | not estimable | (0 studies) | - | No direct evidence identified. |
| Side effects: bleeding | 0 per 1,000 | **0 per 1,000** (0 to 0) | not estimable | (0 studies) | - | No direct evidence identified. |
| Side effects: pain | 987 per 1,000 | **987 per 1,000** (918 to 1,000) | **RR 1.00** (0.93 to 1.07) | 1501 (1 RCT) ^2^ | ⨁⨁◯◯ LOW | Our confidence in the direct estimate is limited. The true effect may be substantially different from the estimate of the effect. |
| Side effects: vomiting | 256 per 1,000 | **358 per 1,000** (291 to 440) | **RR 1.40** (1.14 to 1.72) | 1501 (1 RCT) ^1^ | ⨁⨁◯◯ LOW | Our confidence in the direct estimate is limited. The true effect may be substantially different from the estimate of the effect. |
| Satisfaction | 936 per 1,000 | **927 per 1,000** (861 to 1,000) | **RR 0.99** (0.92 to 1.07) | 1475 (1 RCT) ^1^ | ⨁⨁◯◯ LOW | Our confidence in the direct estimate is limited. The true effect may be substantially different from the estimate of the effect. |
| ***The risk in the intervention group** (and its 95% confidence interval) is based on the assumed risk in the comparison group and the **relative effect** of the intervention (and its 95% CI).   **CI:** Confidence interval; **RR:** Risk ratio. **Studies:** vonHertzen 2010^1^ | | | | | | |

1. **Misoprostol vaginal 400 μg compared with 800 μg in combined regimen**

| **Outcomes** | **Anticipated absolute effects^*^ (95% CI)** | | **Relative effect (95% CI)** | **№ of participants  (studies)** | **GRADE** | **Comments** |
| --- | --- | --- | --- | --- | --- | --- |
|  | **Risk with 800 μg vaginal misoprostol in combined regimen** | **Risk with 400 μg vaginal misoprostol in combined regimen** |  |  |  |  |
| Efficacy: ongoing pregnancy | 11 per 1,000 | **24 per 1,000** (11 to 55) | **RR 2.23** (0.98 to 5.11) | 1482 (1 RCT) ^1^ | ⨁⨁⨁◯ MODERATE | There is probably no difference in ongoing pregnancy when a dose of 800 μg versus 400 μg misoprostol is used vaginally. |
| Efficacy: completed without surgical intervention | 945 per 1,000 | **917 per 1,000** (850 to 992) | **RR 0.97** (0.90 to 1.05) | 1482 (1 RCT) ^1^ | ⨁⨁⨁◯ MODERATE | There is probably no difference in the outcome when a dose of 400 μg or 800 μg misoprostol is used vaginally |
| Safety: serious adverse events and complications | 0 per 1,000 | **0 per 1,000** (0 to 0) | not estimable | (0 studies) | - | No direct evidence identified. |
| Expulsion time | 0 per 1,000 | **0 per 1,000** (0 to 0) | not estimable | (0 studies) | - | No direct evidence identified. |
| Side effects: bleeding | 0 per 1,000 | **0 per 1,000** (0 to 0) | not estimable | (0 studies) | - | No direct evidence identified. |
| Side effects: pain | 981 per 1,000 | **972 per 1,000** (903 to 1,000) | **RR 0.99** (0.92 to 1.07) | 1499 (1 RCT) ^1^ | ⨁⨁◯◯ LOW | Our confidence in the direct estimate is limited. The true effect may be substantially different from the estimate of the effect. |
| Side effects: vomiting | 169 per 1,000 | **142 per 1,000** (112 to 183) | **RR 0.84** (0.66 to 1.08) | 1499 (1 RCT) ^1^ | ⨁⨁◯◯ LOW | Our confidence in the direct estimate is limited. The true effect may be substantially different from the estimate of the effect. |
| Satisfaction | 946 per 1,000 | **937 per 1,000** (870 to 1,000) | **RR 0.99** (0.92 to 1.07) | 1479 (1 RCT) ^1^ | ⨁⨁◯◯ LOW | Our confidence in the direct estimate is limited. The true effect may be substantially different from the estimate of the effect. |
| ***The risk in the intervention group** (and its 95% confidence interval) is based on the assumed risk in the comparison group and the **relative effect** of the intervention (and its 95% CI).   **CI:** Confidence interval; **RR:** Risk ratio. **Studies:** vonHertzen 2010^1^ | | | | | | |

1. **Misoprostol oral 400 μg compared with 600 μg in combined regimen**

| **Outcomes** | **Anticipated absolute effects^*^ (95% CI)** | | **Relative effect (95% CI)** | **№ of participants  (studies)** | **GRADE** | **Comments** |
| --- | --- | --- | --- | --- | --- | --- |
|  | **Risk with 600 μg oral misoprostol in combined regimen** | **Risk with 400 μg oral misoprostol in combined regimen** |  |  |  |  |
| Efficacy: ongoing pregnancy | 3 per 1,000 | **1 per 1,000** (0 to 25) | **RR 0.33** (0.01 to 8.10) | 638 (1 RCT) ^1^ | ⨁⨁◯◯ LOW | Our confidence in the direct estimate is limited. The true effect may be substantially different from the estimate of the effect. |
| Efficacy: completed without surgical intervention | 928 per 1,000 | **937 per 1,000** (844 to 1,000) | **RR 1.01** (0.91 to 1.13) | 638 (1 RCT) ^1^ | ⨁⨁◯◯ LOW | Our confidence in the direct estimate is limited. The true effect may be substantially different from the estimate of the effect. |
| Safety: serious adverse events and complications | 3 per 1,000 | **1 per 1,000** (0 to 25) | **RR 0.33** (0.01 to 8.10) | 638 (1 RCT) ^1^ | ⨁⨁◯◯ LOW | Our confidence in the direct estimate is limited. The true effect may be substantially different from the estimate of the effect. |
| Expulsion time | 0 per 1,000 | **0 per 1,000** (0 to 0) | not estimable | (0 studies) | - | No direct evidence identified |
| Side effects: bleeding | 0 per 1,000 | **0 per 1,000** (0 to 0) | not estimable | (0 studies) | - | No direct evidence identified. |
| Side effects: pain | 0 per 1,000 | **0 per 1,000** (0 to 0) | not estimable | (0 studies) | - | No direct evidence identified. |
| Side effects: vomiting | 236 per 1,000 | **200 per 1,000** (146 to 271) | **RR 0.85** (0.62 to 1.15) | 637 (1 RCT) ^1^ | ⨁⨁◯◯ LOW | Our confidence in the direct estimate is limited. The true effect may be substantially different from the estimate of the effect. |
| Satisfaction | 881 per 1,000 | **899 per 1,000** (802 to 1,000) | **RR 1.02** (0.91 to 1.16) | 599 (1 RCT) ^1^ | ⨁⨁◯◯ LOW | Our confidence in the direct estimate is limited. The true effect may be substantially different from the estimate of the effect. |
| ***The risk in the intervention group** (and its 95% confidence interval) is based on the assumed risk in the comparison group and the **relative effect** of the intervention (and its 95% CI).   **CI:** Confidence interval; **RR:** Risk ratio. **Studies:** Shannon 2006^1^ | | | | | | |

**Table S3. Comparison of dosing intervals between mifepristone and misoprostol in combined regimen**

1. **Misoprostol 800 μg vaginal given < 8 hours compared with > 24 hours after mifepristone**

| **Outcomes** | **Anticipated absolute effects^*^ (95% CI)** | | **Relative effect (95% CI)** | **№ of participants  (studies)** | **GRADE** | **Comments** |
| --- | --- | --- | --- | --- | --- | --- |
|  | **Risk with 800 μg vaginal misoprostol given > 24 hours after mifepristone** | **Risk with 800 μg vaginal misoprostol given < 8 hours after mifepristone** |  |  |  |  |
| Efficacy: ongoing pregnancy (Efficacy) | 5 per 1,000 | **12 per 1,000** (4 to 38) | **RR 2.23** (0.69 to 7.20) | 1525 (4 RCTs) ^1,2^ | ⨁⨁⨁◯ MODERATE | 800 μg misoprostol vaginally administered within 8 hours as compared to after 24 hours probably does not affect the outcome. |
| Efficacy: completed without surgical intervention | 967 per 1,000 | **948 per 1,000** (880 to 1,000) | **RR 0.98** (0.91 to 1.06) | 1525 (2 RCTs) ^1,2^ | ⨁⨁⨁◯ MODERATE | 800 μg misoprostol vaginally administered within 8 hours as compared to after 24 hours probably does not affect the outcome. |
| Safety: serious adverse events and complications | 0 per 1,000 | **0 per 1,000** (0 to 0) | **RR 0.99** (0.02 to 49.60) | 1100 (1 RCT) ^1^ | ⨁⨁⨁◯ MODERATE | 800 μg misoprostol vaginally administered within 8 hours as compared to after 24 hours probably does not affect the outcome. |
| Expulsion time | 0 per 1,000 | **0 per 1,000** (0 to 0) | not estimable | (0 studies) | - | No direct evidence identified. |
| Side effects: bleeding | 0 per 1,000 | **0 per 1,000** (0 to 0) | not estimable | (0 studies) | - | No direct evidence identified. |
| Side effects: pain | 0 per 1,000 | **0 per 1,000** (0 to 0) | not estimable | (0 studies) | - | No direct evidence identified. |
| Side effects: vomiting | 272 per 1,000 | **283 per 1,000** (236 to 337) | **RR 1.04** (0.87 to 1.24) | 1446 (2 RCTs) ^1,2^ | ⨁⨁⨁◯ MODERATE | 800 μg misoprostol vaginally administered within 8 hours as compared to after 24 hours probably does not affect the outcome. |
| Satisfaction | 977 per 1,000 | **996 per 1,000** (850 to 1,000) | **RR 1.02** (0.87 to 1.18) | 357 (1 RCT) ^2^ | ⨁⨁◯◯ LOW | Our confidence in the direct estimate is limited. The true effect may be substantially different from the estimate of the effect. |
| ***The risk in the intervention group** (and its 95% confidence interval) is based on the assumed risk in the comparison group and the **relative effect** of the intervention (and its 95% CI).   **CI:** Confidence interval; **RR:** Risk ratio. **Studies:** Creinin 2007^1^ and Guest 2007^2^ | | | | | | |

1. **Misoprostol 400-800 μg vaginal given 24 hours compared with 48 hours after mifepristone**

| **Outcomes** | **Anticipated absolute effects^*^ (95% CI)** | | **Relative effect (95% CI)** | **№ of participants  (studies)** | **GRADE** | **Comments** |
| --- | --- | --- | --- | --- | --- | --- |
|  | **Risk with 400-800 μg vaginal misoprostol given 48 hours after mifepristone** | **Risk with 400-800 μg vaginal misoprostol given 24 hours after mifepristone** |  |  |  |  |
| Efficacy: ongoing pregnancy | 8 per 1,000 | **7 per 1,000** (3 to 16) | **RR 0.92** (0.40 to 2.12) | 3301 (3 RCTs) ^1,2,3^ | ⨁◯◯◯ VERY LOW | We are uncertain about the effect on this outcome because the certainty of the evidence is very low. |
| Efficacy: completed without surgical intervention | 940 per 1,000 | **931 per 1,000** (752 to 1,000) | **RR 0.99** (0.80 to 1.23) | 192 (3 RCTs) ^1,2,3^ | ⨁◯◯◯ VERY LOW | We are uncertain about the effect on this outcome because the certainty of the evidence is very low. |
| Safety: serious adverse events and complications | 0 per 1,000 | **0 per 1,000** (0 to 0) | not estimable | (0 studies) | - | No direct evidence identified |
| Expulsion time | 0 per 1,000 | **0 per 1,000** (0 to 0) | not estimable | (0 studies) | - | No direct evidence identified |
| Side effects: bleeding | 23 per 1,000 | **22 per 1,000** (3 to 154) | **RR 0.98** (0.14 to 6.79) | 178 (1 RCT) ^1^ | ⨁◯◯◯ VERY LOW | We are uncertain about the effect on this outcome because the certainty of the evidence is very low. |
| Side effects: pain | 0 per 1,000 | **0 per 1,000** (0 to 0) | not estimable | (0 studies) | - | No direct evidence identified |
| Side effects: vomiting | 211 per 1,000 | **195 per 1,000** (169 to 220) | **RR 0.92** (0.80 to 1.04) | 344 (3 RCTs) ^1,2,3^ | ⨁◯◯◯ VERY LOW | We are uncertain about the effect on this outcome because the certainty of the evidence is very low. |
| Satisfaction | 0 per 1,000 | **0 per 1,000** (0 to 0) | not estimable | (0 studies) | - | No direct evidence identified |
| ***The risk in the intervention group** (and its 95% confidence interval) is based on the assumed risk in the comparison group and the **relative effect** of the intervention (and its 95% CI).   **CI:** Confidence interval; **RR:** Risk ratio. **Studies:** Verma 2011^1^, Schaff 2000^2^ and Von Hertzen 2009^3^ | | | | | | |

1. **Misoprostol 400 μg vaginal given concurrently compared with 24 hours after mifepristone**

| **Outcomes** | **Anticipated absolute effects^*^ (95% CI)** | | **Relative effect (95% CI)** | **№ of participants  (studies)** | **GRADE** | **Comments** |
| --- | --- | --- | --- | --- | --- | --- |
|  | **Risk with 400 μg vaginal misoprostol given 24 hours after mifepristone** | **Risk with 400 μg vaginal misoprostol given concurrently with mifepristone** |  |  |  |  |
| Efficacy: ongoing pregnancy | 0 per 1,000 | **0 per 1,000** (0 to 0) | **RR 0.98** (0.02 to 49.25) | 258 (2 RCT) ^1,2^ | ⨁◯◯◯ VERY LOW | We are uncertain about the effect on this outcome because the certainty of the evidence is very low. |
| Efficacy: completed without surgical intervention | 957 per 1,000 | **967 per 1,000** (804 to 1,000) | **RR 1.01** (0.84 to 1.21) | 280 (2 RCT) ^1,2^ | ⨁◯◯◯ VERY LOW | We are uncertain about the effect on this outcome because the certainty of the evidence is very low. |
| Safety: serious adverse events and complications | 0 per 1,000 | **0 per 1,000** (0 to 0) | **RR 1.00** (0.02 to 50.01) | 178 (2 RCT) ^1,2^ | ⨁◯◯◯ VERY LOW | We are uncertain about the effect on this outcome because the certainty of the evidence is very low. |
| Expulsion time | 0 per 1,000 | **0 per 1,000** (0 to 0) | not estimable | (0 studies) | - | No direct evidence identified. |
| Side effects: bleeding | 23 per 1,000 | **22 per 1,000** (3 to 154) | **RR 0.98** (0.14 to 6.79) | 178 (1 RCT) ^1^ | ⨁◯◯◯ VERY LOW | We are uncertain about the effect on this outcome because the certainty of the evidence is very low. |
| Side effects: pain | 50 per 1,000 | **74 per 1,000** (13 to 417) | **RR 1.47** (0.25 to 8.33) | 80 ( RCTs) ^2^ | ⨁◯◯◯ VERY LOW | We are uncertain about the effect on this outcome because the certainty of the evidence is very low. |
| Side effects: vomiting | 141 per 1,000 | **110 per 1,000** (58 to 214) | **RR 0.78** (0.41 to 1.52) | 258 (2 RCTs) ^1,2^ | ⨁◯◯◯ VERY LOW | We are uncertain about the effect on this outcome because the certainty of the evidence is very low. |
| Satisfaction | 950 per 1,000 | **969 per 1,000** (703 to 1,000) | **RR 1.02** (0.74 to 1.39) | 80 (1 RCT) ^2^ | ⨁◯◯◯ VERY LOW | We are uncertain about the effect on this outcome because the certainty of the evidence is very low. |
| ***The risk in the intervention group** (and its 95% confidence interval) is based on the assumed risk in the comparison group and the **relative effect** of the intervention (and its 95% CI).   **CI:** Confidence interval; **RR:** Risk ratio. **Studies:** Verma 2017^1^ and Goel 2011^2^ | | | | | | |

1. **Misoprostol 400 μg oral given < 8 hours compared with 48 hours after mifepristone**

| **Outcomes** | **Anticipated absolute effects^*^ (95% CI)** | | **Relative effect (95% CI)** | **№ of participants  (studies)** | **GRADE** | **Comments** |
| --- | --- | --- | --- | --- | --- | --- |
|  | **Risk with 400 μg oral misoprostol given 48 hours after mifepristone** | **Risk with 400 μg oral misoprostol given < 8 hours after mifepristone** |  |  |  |  |
| Efficacy: ongoing pregnancy (Efficacy) | 0 per 1,000 | **0 per 1,000** (0 to 0) | **RR 8.34** (0.46 to 151.20) | 100 (1 RCT) ^1^ | ⨁◯◯◯ VERY LOW | We are uncertain about the effect on this outcome because the certainty of the evidence is very low. |
| Efficacy: completed without surgical intervention | 900 per 1,000 | **819 per 1,000** (594 to 1,000) | **RR 0.91** (0.66 to 1.25) | 100 (1 RCT) ^1^ | ⨁◯◯◯ VERY LOW | We are uncertain about the effect on this outcome because the certainty of the evidence is very low. |
| Safety: serious adverse events and complications | 20 per 1,000 | **39 per 1,000** (4 to 418) | **RR 1.96** (0.18 to 20.90) | 100 (1 RCT) ^1^ | ⨁◯◯◯ VERY LOW | We are uncertain about the effect on this outcome because the certainty of the evidence is very low. |
| Expulsion time | 0 per 1,000 | **0 per 1,000** (0 to 0) | not estimable | (0 studies) | - | No direct evidence identified. |
| Side effects: bleeding | 0 per 1,000 | **0 per 1,000** (0 to 0) | not estimable | (0 studies) | - | No direct evidence identified. |
| Side effects: pain | 0 per 1,000 | **0 per 1,000** (0 to 0) | not estimable | (0 studies) | - | No direct evidence identified. |
| Side effects: vomiting | 0 per 1,000 | **0 per 1,000** (0 to 0) | not estimable | (0 studies) | - | No direct evidence identified. |
| Satisfaction | 0 per 1,000 | **0 per 1,000** (0 to 0) | not estimable | (0 studies) | - | No direct evidence identified. |
| ***The risk in the intervention group** (and its 95% confidence interval) is based on the assumed risk in the comparison group and the **relative effect** of the intervention (and its 95% CI).   **CI:** Confidence interval; **RR:** Risk ratio. **Studies:** Tendler 2015^1^ | | | | | | |

**Table S4. Comparison of misoprostol routes in combined mifepristone-misoprostol regimen**

1. **Misoprostol 400 μg sublingual compared with vaginal in combined regimen**

| **Outcomes** | **Anticipated absolute effects^*^ (95% CI)** | | **Relative effect (95% CI)** | **№ of participants  (studies)** | **GRADE** | **Comments** |
| --- | --- | --- | --- | --- | --- | --- |
|  | **Risk with 400 μg vaginal misoprostol in combined regimen** | **Risk with 400 μg sublingual misoprostol in combined regimen** |  |  |  |  |
| Efficacy: ongoing pregnancy | 24 per 1,000 | **19 per 1,000** (10 to 38) | **RR 0.79** (0.39 to 1.55) | 1479 (1 RCT) ^1^ | ⨁⨁⨁◯ MODERATE | There is probably no difference in ongoing pregnancy rates when the misoprostol is administered PV versus SL. |
| Efficacy: completed without surgical intervention | 896 per 1,000 | **905 per 1,000** (842 to 976) | **RR 1.01** (0.94 to 1.09) | 1479 (1 RCT) ^1^ | ⨁⨁⨁◯ MODERATE | There is probably no difference in need for a surgery to complete the abortion when the misoprostol is administered PV vs SL |
| Safety: serious adverse events and complications | 0 per 1,000 | **0 per 1,000** (0 to 0) | not estimable | (0 studies) | - | There is probably no difference in SAE when the misoprostol is administered PV at 24 hours versus 48 hours or later. |
| Expulsion time | 0 per 1,000 | **0 per 1,000** (0 to 0) | not estimable | (0 studies) | - | No direct evidence identified |
| Side effects: bleeding | 0 per 1,000 | **0 per 1,000** (0 to 0) | not estimable | (0 studies) | - | No direct evidence identified. |
| Side effects: pain | 960 per 1,000 | **970 per 1,000** (893 to 1,000) | **RR 1.01** (0.93 to 1.08) | 1499 (1 RCT) ^1^ | ⨁⨁⨁◯ MODERATE | There is probably no difference in pain when the misoprostol is administered PV versus SL. |
| Side effects: vomiting | 140 per 1,000 | **185 per 1,000** (147 to 234) | **RR 1.32** (1.05 to 1.67) | 1499 (1 RCT) ^1^ | ⨁⨁⨁◯ MODERATE | There is probably no difference in emesis when the misoprostol is administered PV versus SL. |
| Satisfaction | 936 per 1,000 | **936 per 1,000** (880 to 1,000) | **RR 1.00** (0.94 to 1.07) | 1473 (1 RCT) ^1^ | ⨁⨁⨁◯ MODERATE | There is probably no difference in satisfaction when the misoprostol is administered PV versus SL. |
| ***The risk in the intervention group** (and its 95% confidence interval) is based on the assumed risk in the comparison group and the **relative effect** of the intervention (and its 95% CI).   **CI:** Confidence interval; **RR:** Risk ratio. **Studies:** VonHertzen 2010^1^ | | | | | | |

1. **Misoprostol 800 μg vaginal compared with sublingual in combined regimen**

| **Outcomes** | **Anticipated absolute effects^*^ (95% CI)** | | **Relative effect (95% CI)** | **№ of participants  (studies)** | **GRADE** | **Comments** |
| --- | --- | --- | --- | --- | --- | --- |
|  | **Risk with 800 μg sublingual misoprostol in combined regimen** | **Risk with 800 μg vaginal misoprostol in combined regimen** |  |  |  |  |
| Efficacy: ongoing pregnancy | 11 per 1,000 | **5 per 1,000** (2 to 18) | **RR 0.50** (0.15 to 1.67) | 1483 (1 RCT) ^1^ | ⨁⨁⨁◯ MODERATE ^a^ | There is probably no difference in the outcome when misoprostol is administered vaginally vs sublingually. |
| Efficacy: completed without surgical intervention | 945 per 1,000 | **935 per 1,000** (869 to 1,000) | **RR 0.99** (0.92 to 1.07) | 1483 (1 RCT) ^1^ | ⨁⨁⨁◯ MODERATE ^a^ | There is probably no difference in the outcome when misoprostol is administered vaginally vs sublingually. |
| Safety: serious adverse events and complications | 0 per 1,000 | **0 per 1,000** (0 to 0) | not estimable | (0 studies) ^b^ | - | No direct evidence identified. |
| Expulsion time | 0 per 1,000 | **0 per 1,000** (0 to 0) | not estimable | (0 studies) | - | No direct evidence identified. |
| Side effects: bleeding | 0 per 1,000 | **0 per 1,000** (0 to 0) | not estimable | (0 studies) | - | No direct evidence identified. |
| Side effects: pain | 981 per 1,000 | **981 per 1,000** (913 to 1,000) | **RR 1.00** (0.93 to 1.07) | 1501 ( RCTs) ^2,3^ | ⨁⨁◯◯ LOW ^a,c^ | Our confidence in the direct estimate is limited. The true effect may be substantially different from the estimate of the effect. |
| Side effects: vomiting | 169 per 1,000 | **237 per 1,000** (193 to 291) | **RR 1.40** (1.14 to 1.72) | 1501 (1 RCT) ^1^ | ⨁⨁◯◯ LOW ^a,c^ | Our confidence in the direct estimate is limited. The true effect may be substantially different from the estimate of the effect. |
| Satisfaction | 946 per 1,000 | **937 per 1,000** (870 to 1,000) | **RR 0.99** (0.92 to 1.07) | 1481 (1 RCT) ^1^ | ⨁◯◯◯ VERY LOW ^a,c^ | We are uncertain about the effect on this outcome because the certainty of the evidence is very low. |
| ***The risk in the intervention group** (and its 95% confidence interval) is based on the assumed risk in the comparison group and the **relative effect** of the intervention (and its 95% CI).   **CI:** Confidence interval; **RR:** Risk ratio. **Studies:** VonHertzen 2010^1^ | | | | | | |

1. **Misoprostol 600/800 μg sublingual compared with 800 μg vaginal in combined regimen**

| **Outcomes** | **Anticipated absolute effects^*^ (95% CI)** | | **Relative effect (95% CI)** | **№ of participants  (studies)** | **GRADE** | **Comments** |
| --- | --- | --- | --- | --- | --- | --- |
|  | **Risk with 800 μg vaginal misoprostol in combined regimen** | **Risk with 600/800 μg sublingual misoprostol in combined regimen** |  |  |  |  |
| Efficacy: ongoing pregnancy | 17 per 1,000 | **2 per 1,000** (1 to 51) | **RR 0.15** (0.08 to 3.05) | 346 (2 RCTs) ^1,2^ | ⨁⨁◯◯ LOW | Our confidence in the direct estimate is limited. The true effect may be substantially different from the estimate of the effect. |
| Efficacy: completed without surgical intervention | 956 per 1,000 | **965 per 1,000** (832 to 1,000) | **RR 1.01** (0.87 to 1.18) | 346 (2 RCTs) ^1,2^ | ⨁⨁◯◯ LOW | Our confidence in the direct estimate is limited. The true effect may be substantially different from the estimate of the effect. |
| Safety: serious adverse events and complications | 0 per 1,000 | **0 per 1,000** (0 to 0) | **RR 1.00** (0.02 to 49.96) | 224 (1 RCTs) ^1^ | ⨁⨁◯◯ LOW | Our confidence in the direct estimate is limited. The true effect may be substantially different from the estimate of the effect. |
| Expulsion time | 0 per 1,000 | **0 per 1,000** (0 to 0) | not estimable | (0 studies) | - | No direct evidence identified |
| Side effects: bleeding | 0 per 1,000 | **0 per 1,000** (0 to 0) | not estimable | (0 studies) | - | No direct evidence identified. |
| Side effects: pain | 964 per 1,000 | **974 per 1,000** (810 to 1,000) | **RR 1.01** (0.84 to 1.22) | 224 (1 RCT) ^1^ | ⨁⨁⨁◯ MODERATE | There is probably no difference in pain when the misoprostol is administered PV versus SL. |
| Side effects: vomiting | 964 per 1,000 | **521 per 1,000** (386 to 704) | **RR 0.54** (0.40 to 0.73) | 224 (1 RCT) ^1^ | ⨁⨁⨁◯ MODERATE | There is probably no difference in emesis when the misoprostol is administered PV versus SL. |
| Satisfaction | 0 per 1,000 | **0 per 1,000** (0 to 0) | not estimable | ( 0 studies) | - | No direct evidence identified. |
| ***The risk in the intervention group** (and its 95% confidence interval) is based on the assumed risk in the comparison group and the **relative effect** of the intervention (and its 95% CI).   **CI:** Confidence interval; **RR:** Risk ratio. **Studies:** Tang 2003^1^ and Hamoda 2005^2^ | | | | | | |

1. **Misoprostol 800 μg oral compared with vaginal in combined regimen**

| **Outcomes** | **Anticipated absolute effects^*^ (95% CI)** | | **Relative effect (95% CI)** | **№ of participants  (studies)** | **GRADE** | **Comments** |
| --- | --- | --- | --- | --- | --- | --- |
|  | **Risk with 800 μg vaginal misoprostol in combined regimen** | **Risk with 800 μg oral misoprostol in combined regimen** |  |  |  |  |
| Efficacy: ongoing pregnancy (Efficacy) | 1 per 1,000 | **9 per 1,000** (3 to 33) | **RR 6.70** (1.88 to 23.86) | 1287 (3 RCTs) ^1,2,3^ | ⨁⨁⨁◯ MODERATE | 800 mg misoprostol orally administered probably slightly increases the risk of an ongoing pregnancy. |
| Efficacy: completed without surgical intervention | 985 per 1,000 | **926 per 1,000** (837 to 1,000) | **RR 0.94** (0.85 to 1.04) | 1455 (3 RCTs) ^1,2,3^ | ⨁⨁⨁◯ MODERATE | 800 mg misoprostol vaginally administered probably does not affect the need for surgical intervention. |
| Safety: serious adverse events and complications | 8 per 1,000 | **3 per 1,000** (0 to 63) | **RR 0.35** (0.01 to 8.35) | 263 (1 RCT) ^2^ | ⨁◯◯◯ VERY LOW | We are uncertain about the effect on this outcome because the certainty of the evidence is very low. |
| Expulsion time | 932 per 1,000 | **848 per 1,000** (699 to 1,000) | **RR 0.91** (0.75 to 1.10) | 263 (1 RCT) ^2^ | ⨁◯◯◯ VERY LOW | We are uncertain about the effect on this outcome because the certainty of the evidence is very low. |
| Side effects: bleeding | 0 per 1,000 | **0 per 1,000** (0 to 0) | not estimable | (0 studies) | - | No direct evidence identified. |
| Side effects: pain | 2 per 1,000 | **5 per 1,000** (1 to 52) | **RR 3.25** (0.34 to 31.15) | 1144 (1 RCTs) ^1^ | ⨁⨁⨁◯ MODERATE | 800 mg misoprostol vaginally administered probably does not affect the outcome. |
| Side effects: vomiting | 356 per 1,000 | **306 per 1,000** (260 to 363) | **RR 0.86** (0.73 to 1.02) | 1219 (2 RCTs) ^1,2^ | ⨁⨁⨁◯ MODERATE | 800 mg misoprostol vaginally administered probably does not affect the outcome. |
| Satisfaction | 0 per 1,000 | **0 per 1,000** (0 to 0) | not estimable | (0 studies) | - | No direct evidence |
| ***The risk in the intervention group** (and its 95% confidence interval) is based on the assumed risk in the comparison group and the **relative effect** of the intervention (and its 95% CI).   **CI:** Confidence interval; **RR:** Risk ratio. **Studies:** Schaff 2001^1^, el-Refaey 1995^2^ and Schaff 2002^3^ | | | | | | |

1. **Misoprostol 400 μg oral compared with 800 μg vaginal in combined regimen**

| **Outcomes** | **Anticipated absolute effects^*^ (95% CI)** | | **Relative effect (95% CI)** | **№ of participants  (studies)** | **GRADE** | **Comments** |
| --- | --- | --- | --- | --- | --- | --- |
|  | **Risk with 800 μg vaginal misoprostol in combined regimen** | **Risk with 400 μg oral misoprostol in combined regimen** |  |  |  |  |
| Efficacy: ongoing pregnancy | 2 per 1,000 | **4 per 1,000** (1 to 26) | **RR 2.38** (0.34 to 16.81) | 1378 (2 RCTs) ^1,2^ | ⨁⨁⨁◯ MODERATE | There is probably no difference in ongoing pregnancy rates when misoprostol is given orally versus vaginally. |
| Efficacy: completed without surgical intervention | 970 per 1,000 | **951 per 1,000** (883 to 1,000) | **RR 0.98** (0.91 to 1.04) | 2025 (2 RCTs) ^1,2^ | ⨁⨁⨁◯ MODERATE | There is probably no difference in need for surgical intervention when misoprostol is given orally versus vaginally. |
| Safety: serious adverse events and complications | 3 per 1,000 | **1 per 1,000** (0 to 26) | **RR 0.33** (0.01 to 8.15) | 637 (1 RCT) ^1^ | ⨁⨁◯◯ LOW | Our confidence in the direct estimate is limited. The true effect may be substantially different from the estimate of the effect. |
| Expulsion time | 0 per 1,000 | **0 per 1,000** (0 to 0) | not estimable | (0 studies) | - | No direct evidence identified |
| Side effects: bleeding | 8 per 1,000 | **40 per 1,000** (12 to 52) | **RR 5.19** (1.61 to 6.79) | 741 (1 RCT) ^2^ | ⨁⨁◯◯ LOW | Our confidence in the direct estimate is limited. The true effect may be substantially different from the estimate of the effect. |
| Side effects: pain | 958 per 1,000 | **900 per 1,000** (804 to 1,000) | **RR 0.94** (0.84 to 1.07) | 738 (1 RCT) ^2^ | ⨁⨁◯◯ LOW | Our confidence in the direct estimate is limited. The true effect may be substantially different from the estimate of the effect. |
| Side effects: vomiting | 236 per 1,000 | **361 per 1,000** (252 to 519) | **RR 1.53** (1.07 to 2.20) | 637 (1 RCT) ^1^ | ⨁⨁◯◯ LOW | Our confidence in the direct estimate is limited. The true effect may be substantially different from the estimate of the effect. |
| Satisfaction | 881 per 1,000 | **899 per 1,000** (802 to 1,000) | **RR 1.02** (0.91 to 1.16) | 599 (1 RCT) ^1^ | ⨁⨁◯◯ LOW | Our confidence in the direct estimate is limited. The true effect may be substantially different from the estimate of the effect. |
| ***The risk in the intervention group** (and its 95% confidence interval) is based on the assumed risk in the comparison group and the **relative effect** of the intervention (and its 95% CI).   **CI:** Confidence interval; **RR:** Risk ratio. **Studies:** Shannon 2006^1^ and Schaff 2002^2^ | | | | | | |

1. **Misoprostol 800 μg buccal compared with sublingual in combined regimen**

| **Outcomes** | **Anticipated absolute effects^*^ (95% CI)** | | **Relative effect (95% CI)** | **№ of participants  (studies)** | **GRADE** | **Comments** |
| --- | --- | --- | --- | --- | --- | --- |
|  | **Risk with 800 μg sublingual misoprostol in combined regimen** | **Risk with 800 μg buccal misoprostol in combined regimen** |  |  |  |  |
| Efficacy: ongoing pregnancy | 22 per 1,000 | **22 per 1,000** (0 to 1,000) | **RR 0.98** (0.02 to 49.25) | 90 (1 RCT) ^1^ | ⨁◯◯◯ VERY LOW | We are uncertain about the effect on this outcome because the certainty of the evidence is very low. |
| Efficacy: completed without surgical intervention | 978 per 1,000 | **958 per 1,000** (714 to 1,000) | **RR 0.98** (0.73 to 1.33) | 90 (1 RCT) ^1^ | ⨁◯◯◯ VERY LOW | We are uncertain about the effect on this outcome because the certainty of the evidence is very low. |
| Safety: serious adverse events and complications | 0 per 1,000 | **0 per 1,000** (0 to 0) | **RR 0.98** (0.02 to 48.70) | 178 (0 RCTs) ^1^ | ⨁◯◯◯ VERY LOW | We are uncertain about the effect on this outcome because the certainty of the evidence is very low. |
| Expulsion time | 0 per 1,000 | **0 per 1,000** (0 to 0) | not estimable | (0 studies) | - |  |
| Side effects: bleeding | 0 per 1,000 | **0 per 1,000** (0 to 0) | not estimable | (0 studies) | - |  |
| Side effects: pain | 0 per 1,000 | **0 per 1,000** (0 to 0) | not estimable | (0 studies) | - |  |
| Side effects: vomiting | 141 per 1,000 | **110 per 1,000** (58 to 214) | **RR 0.78** (0.41 to 1.52) | 258 (2 RCTs) ^1^ | ⨁◯◯◯ VERY LOW | We are uncertain about the effect on this outcome because the certainty of the evidence is very low. |
| Satisfaction | 0 per 1,000 | **0 per 1,000** (0 to 0) | not estimable | (0 studies) | - |  |
| ***The risk in the intervention group** (and its 95% confidence interval) is based on the assumed risk in the comparison group and the **relative effect** of the intervention (and its 95% CI).   **CI:** Confidence interval; **RR:** Risk ratio. **Studies:** Chai 2013^1^ | | | | | | |

1. **Misoprostol 400 μg buccal compared with sublingual in combined regimen**

| **Outcomes** | **Anticipated absolute effects^*^ (95% CI)** | | **Relative effect (95% CI)** | **№ of participants  (studies)** | **GRADE** | **Comments** |
| --- | --- | --- | --- | --- | --- | --- |
|  | **Risk with 400 μg sublingual misoprostol in combined regimen** | **Risk with 400 μg buccal misoprostol in combined regimen** |  |  |  |  |
| Efficacy: ongoing pregnancy | 15 per 1,000 | **23 per 1,000** (3 to 165) | **RR 1.55** (0.22 to 11.03) | 539 (1 RCTs) ^1^ | ⨁⨁◯◯ LOW | Our confidence in the direct estimate is limited. The true effect may be substantially different from the estimate of the effect. |
| Efficacy: completed without surgical intervention | 974 per 1,000 | **954 per 1,000** (886 to 1,000) | **RR 0.98** (0.91 to 1.04) | 539 (1 RCTs) ^1^ | ⨁⨁◯◯ LOW | Our confidence in the direct estimate is limited. The true effect may be substantially different from the estimate of the effect. |
| Safety: serious adverse events and complications | 0 per 1,000 | **0 per 1,000** (0 to 0) | **RR 0.33** (0.01 to 8.15) | 539 (1 RCT) ^1^ | ⨁⨁◯◯ LOW | Our confidence in the direct estimate is limited. The true effect may be substantially different from the estimate of the effect. |
| Expulsion time | 0 per 1,000 | **0 per 1,000** (0 to 0) | not estimable | (0 studies) | - | No direct evidence identified |
| Side effects: bleeding | 562 per 1,000 | **1000 per 1,000** (904 to 1,000) | **RR 5.19** (1.61 to 6.79) | 526 (1 RCT) ^1^ | ⨁⨁◯◯ LOW | Our confidence in the direct estimate is limited. The true effect may be substantially different from the estimate of the effect. |
| Side effects: pain | 800 per 1,000 | **752 per 1,000** (672 to 856) | **RR 0.94** (0.84 to 1.07) | 526 (1 RCT) ^1^ | ⨁⨁◯◯ LOW | Our confidence in the direct estimate is limited. The true effect may be substantially different from the estimate of the effect. |
| Side effects: vomiting | 219 per 1,000 | **335 per 1,000** (235 to 482) | **RR 1.53** (1.07 to 2.20) | 526 (1 RCT) ^1^ | ⨁⨁◯◯ LOW | Our confidence in the direct estimate is limited. The true effect may be substantially different from the estimate of the effect. |
| Satisfaction | 958 per 1,000 | **978 per 1,000** (872 to 1,000) | **RR 1.02** (0.91 to 1.16) | 533 (1 RCT) ^1^ | ⨁⨁◯◯ LOW | Our confidence in the direct estimate is limited. The true effect may be substantially different from the estimate of the effect. |
| ***The risk in the intervention group** (and its 95% confidence interval) is based on the assumed risk in the comparison group and the **relative effect** of the intervention (and its 95% CI).   **CI:** Confidence interval; **RR:** Risk ratio. **Studies:** Raghavan 2010^1^ | | | | | | |

1. **Misoprostol 800 μg buccal compared with vaginal in combined regimen**

| **Outcomes** | **Anticipated absolute effects^*^ (95% CI)** | | **Relative effect (95% CI)** | **№ of participants  (studies)** | **GRADE** | **Comments** |
| --- | --- | --- | --- | --- | --- | --- |
|  | **Risk with 800 μg vaginal misoprostol in combined regimen** | **Risk with 800 μg buccal misoprostol in combined regimen** |  |  |  |  |
| Efficacy: ongoing pregnancy | 19 per 1,000 | **9 per 1,000** (2 to 50) | **RR 0.49** (0.09 to 2.68) | 429 (1 RCT) ^1^ | ⨁⨁◯◯ LOW ^a,b^ | Our confidence in the direct estimate is limited. The true effect may be substantially different from the estimate of the effect. |
| Efficacy: completed without surgical intervention | 934 per 1,000 | **934 per 1,000** (813 to 1,000) | **RR 1.00** (0.87 to 1.15) | 429 (1 RCT) | ⨁⨁◯◯ LOW ^a,b^ | Our confidence in the direct estimate is limited. The true effect may be substantially different from the estimate of the effect. |
| Safety: serious adverse events and complications | 0 per 1,000 | **0 per 1,000** (0 to 0) | **RR 2.94** (0.12 to 71.80) | 429 (1 RCT) | ⨁⨁◯◯ LOW ^a,b^ | Our confidence in the direct estimate is limited. The true effect may be substantially different from the estimate of the effect. |
| Expulsion time | 0 per 1,000 | **0 per 1,000** (0 to 0) | not estimable | (0 studies) | - | No direct evidence identified |
| Side effects: bleeding | 0 per 1,000 | **0 per 1,000** (0 to 0) | not estimable | (0 studies) | - | No direct evidence identified |
| Side effects: pain | 0 per 1,000 | **0 per 1,000** (0 to 0) | not estimable | (0 studies) | - | No direct evidence identified |
| Side effects: vomiting | 319 per 1,000 | **354 per 1,000** (271 to 469) | **RR 1.11** (0.85 to 1.47) | 429 (1 RCTs) ^1^ | ⨁⨁◯◯ LOW ^a,b^ | Our confidence in the direct estimate is limited. The true effect may be substantially different from the estimate of the effect. |
| Satisfaction | 948 per 1,000 | **929 per 1,000** (805 to 1,000) | **RR 0.98** (0.85 to 1.13) | 423 (1 RCTs) | ⨁⨁◯◯ LOW ^a,b,c^ | Our confidence in the direct estimate is limited. The true effect may be substantially different from the estimate of the effect. |
| ***The risk in the intervention group** (and its 95% confidence interval) is based on the assumed risk in the comparison group and the **relative effect** of the intervention (and its 95% CI).   **CI:** Confidence interval; **RR:** Risk ratio. **Studies:** Middleton 2005^1^ | | | | | | |

1. **Misoprostol 800 μg oral compared with buccal in combined regimen**

| **Outcomes** | **Anticipated absolute effects^*^ (95% CI)** | | **Relative effect (95% CI)** | **№ of participants  (studies)** | **GRADE** | **Comments** |
| --- | --- | --- | --- | --- | --- | --- |
|  | **Risk with 800 μg buccal misoprostol in combined regimen** | **Risk with 800 μg oral misoprostol in combined regimen** |  |  |  |  |
| Efficacy: ongoing pregnancy | 10 per 1,000 | **34 per 1,000** (11 to 103) | **RR 3.61** (1.20 to 10.80) | 847 (1 RCT) ^1^ | ⨁⨁◯◯ LOW | Our confidence in the direct estimate is limited. The true effect may be substantially different from the estimate of the effect. |
| Efficacy: completed without surgical intervention | 962 per 1,000 | **933 per 1,000** (847 to 1,000) | **RR 0.97** (0.88 to 1.07) | 847 (1 RCT) ^1^ | ⨁⨁◯◯ LOW | Our confidence in the direct estimate is limited. The true effect may be substantially different from the estimate of the effect. |
| Safety: serious adverse events and complications | 2 per 1,000 | **1 per 1,000** (0 to 19) | **RR 0.33** (0.01 to 8.08) | 847 (1 RCT) ^1^ | ⨁⨁◯◯ LOW | Our confidence in the direct estimate is limited. The true effect may be substantially different from the estimate of the effect. |
| Expulsion time | 0 per 1,000 | **0 per 1,000** (0 to 0) | not estimable | (0 studies) | - | No direct evidence identified. |
| Side effects: bleeding | 0 per 1,000 | **0 per 1,000** (0 to 0) | not estimable | (0 studies) | - | No direct evidence identified. |
| Side effects: pain | 0 per 1,000 | **0 per 1,000** (0 to 0) | not estimable | (0 studies) | - | No direct evidence identified. |
| Side effects: vomiting | 476 per 1,000 | **447 per 1,000** (376 to 528) | **RR 0.94** (0.79 to 1.11) | 830 (1 RCT) ^1^ | ⨁⨁◯◯ LOW | Our confidence in the direct estimate is limited. The true effect may be substantially different from the estimate of the effect. |
| Satisfaction | 911 per 1,000 | **929 per 1,000** (829 to 1,000) | **RR 1.02** (0.91 to 1.12) | 835 (1 RCT) ^1^ | ⨁⨁◯◯ LOW | Our confidence in the direct estimate is limited. The true effect may be substantially different from the estimate of the effect. |
| ***The risk in the intervention group** (and its 95% confidence interval) is based on the assumed risk in the comparison group and the **relative effect** of the intervention (and its 95% CI).   **CI:** Confidence interval; **RR:** Risk ratio. **Studies:** Winikoff 2008^1^ | | | | | | |

1. **Misoprostol 400 μg oral compared with sublingual in combined regimen**

| **Outcomes** | **Anticipated absolute effects^*^ (95% CI)** | | **Relative effect (95% CI)** | **№ of participants  (studies)** | **GRADE** | **Comments** |
| --- | --- | --- | --- | --- | --- | --- |
|  | **Risk with 400 μg sublingual misoprostol in combined regimen** | **Risk with 400 μg oral misoprostol in combined regimen** |  |  |  |  |
| Efficacy: ongoing pregnancy (Efficacy) | 18 per 1,000 | **6 per 1,000** (1 to 36) | **RR 0.44** (0.10 to 1.96) | 564 (2 RCT) ^1,2^ | ⨁⨁◯◯ LOW | Our confidence in the direct estimate is limited. The true effect may be substantially different from the estimate of the effect. |
| Efficacy: completed without surgical intervention | 942 per 1,000 | **952 per 1,000** (839 to 1,000) | **RR 1.03** (0.99 to 1.07 | 564 (2 RCTs) ^1,2^ | ⨁⨁◯◯ LOW | Our confidence in the direct estimate is limited. The true effect may be substantially different from the estimate of the effect. |
| Safety: serious adverse events and complications | 0 per 1,000 | **0 per 1,000** (0 to 0) | **RR 0.98** (0.01 to 49.14) | 471 (1 RCT) ^2^ | ⨁⨁◯◯ LOW | Our confidence in the direct estimate is limited. The true effect may be substantially different from the estimate of the effect. |
| Expulsion time | 0 per 1,000 | **0 per 1,000** (0 to 0) | not estimable | (0 studies) | - | No direct evidence identified. |
| Side effects: bleeding | 194 per 1,000 | **204 per 1,000** (140 to 295) | **RR 1.05** (0.72 to 1.52) | 470 (1 RCT) ^2^ | ⨁⨁◯◯ LOW | Our confidence in the direct estimate is limited. The true effect may be substantially different from the estimate of the effect. |
| Side effects: pain | 339 per 1,000 | **336 per 1,000** (261 to 428) | **RR 0.99** (0.77 to 1.26) | 563 (2 RCTs) ^1,2^ | ⨁◯◯◯ VERY LOW | We are uncertain about the effect on this outcome because the certainty of the evidence is very low. |
| Side effects: vomiting | 410 per 1,000 | **447 per 1,000** (328 to 554) | **RR 1.09** (0.80 to 1.35) | 564 (2 RCTs) ^1,2^ | ⨁◯◯◯ VERY LOW | We are uncertain about the effect on this outcome because the certainty of the evidence is very low. |
| Satisfaction | 914 per 1,000 | **932 per 1,000** (813 to 1,000) | **RR 1.02** (0.89 to 1.18) | 470 (1 RCT) ^2^ | ⨁⨁◯◯ LOW | Our confidence in the direct estimate is limited. The true effect may be substantially different from the estimate of the effect. |
| ***The risk in the intervention group** (and its 95% confidence interval) is based on the assumed risk in the comparison group and the **relative effect** of the intervention (and its 95% CI).   **CI:** Confidence interval; **RR:** Risk ratio. **Studies:** Dahiya 2011^1^ and Raghavan 2009^2^ | | | | | | |

**Table S5. Comparison of different misoprostol only regimens**

1. **Misoprostol 400 μg oral every 3 hours for 4 doses compared with 600 μg vaginal misoprostol once**

| **Outcomes** | **Anticipated absolute effects^*^ (95% CI)** | | **Relative effect (95% CI)** | **№ of participants  (studies)** | **GRADE** | **Comments** |
| --- | --- | --- | --- | --- | --- | --- |
|  | **Risk with 600 μg vaginal misoprostol once** | **Risk with 400 μg oral misoprostol every 3 h (for 4 doses)** |  |  |  |  |
| Efficacy: ongoing pregnancy (Efficacy) | 200 per 1,000 | **300 per 1,000** (134 to 660) | **RR 1.50** (0.67 to 3.30) | 76 (1 RCT) ^1^ | ⨁◯◯◯ VERY LOW | We are uncertain about the effect on this outcome because the certainty of the evidence is very low. |
| Efficacy: completed without surgical intervention | 425 per 1,000 | **399 per 1,000** (221 to 722) | **RR 0.94** (0.52 to 1.70) | 76 (1 RCT) ^1^ | ⨁◯◯◯ VERY LOW | We are uncertain about the effect on this outcome because the certainty of the evidence is very low. |
| Safety: serious adverse events and complications | 0 per 1,000 | **0 per 1,000** (0 to 0) | not estimable | (0 studies) | - | No direct evidence identified |
| Expulsion time <24 hours | 0 per 1,000 | **0 per 1,000** (0 to 0) | not estimable | (0 studies) | - | No direct evidence identified |
| Side effects: bleeding | 0 per 1,000 | **0 per 1,000** (0 to 0) | not estimable | (0 studies) | - | No direct evidence identified |
| Side effects: pain | 950 per 1,000 | **941 per 1,000** (684 to 1,000) | **RR 0.99** (0.72 to 1.40) | 76 (1 RCT) ^1^ | ⨁◯◯◯ VERY LOW | We are uncertain about the effect on this outcome because the certainty of the evidence is very low. |
| Side effects: vomiting | 75 per 1,000 | **285 per 1,000** (87 to 930) | **RR 3.80** (1.16 to 12.40) | 76 (1 RCT) ^1^ | ⨁◯◯◯ VERY LOW | We are uncertain about the effect on this outcome because the certainty of the evidence is very low. |
| Satisfaction | 450 per 1,000 | **405 per 1,000** (225 to 720) | **RR 0.9** (0.5 to 1.6) | 76 (1 RCT) ^1^ | ⨁◯◯◯ VERY LOW | No direct evidence identified. |
| ***The risk in the intervention group** (and its 95% confidence interval) is based on the assumed risk in the comparison group and the **relative effect** of the intervention (and its 95% CI).   **CI:** Confidence interval; **RR:** Risk ratio. **Studies:** Blanchard 2005^1^ | | | | | | |

1. **Misoprostol 800 μg oral every 6 hours for 2 doses compared with 600 μg vaginal misoprostol once**

| **Outcomes** | **Anticipated absolute effects^*^ (95% CI)** | | **Relative effect (95% CI)** | **№ of participants  (studies)** | **GRADE** | **Comments** |
| --- | --- | --- | --- | --- | --- | --- |
|  | **Risk with 600 μg vaginal misoprostol once** | **Risk with 800 μg oral misoprostol every 6 h (for 2 doses)** |  |  |  |  |
| Efficacy: ongoing pregnancy (Efficacy) | 200 per 1,000 | **172 per 1,000** (56 to 518) | **RR 0.86** (0.28 to 2.59) | 64 (1 RCT) ^1^ | ⨁◯◯◯ VERY LOW | We are uncertain about the effect on this outcome because the certainty of the evidence is very low. |
| Efficacy: completed without surgical intervention | 425 per 1,000 | **476 per 1,000** (259 to 871) | **RR 1.12** (0.61 to 2.05) | 64 (1 RCT) ^1^ | ⨁◯◯◯ VERY LOW | We are uncertain about the effect on this outcome because the certainty of the evidence is very low. |
| Safety: serious adverse events and complications | 0 per 1,000 | **0 per 1,000** (0 to 0) | not estimable | (0 studies) | - | No direct evidence identified |
| Expulsion time <24 hours | 0 per 1,000 | **0 per 1,000** (0 to 0) | not estimable | (0 studies) | - | No direct evidence identified |
| Side effects: bleeding | 0 per 1,000 | **0 per 1,000** (0 to 0) | not estimable | (0 studies) | - | No direct evidence identified |
| Side effects: pain | 950 per 1,000 | **950 per 1,000** (656 to 1,000) | **RR 1.00** (0.69 to 1.45) | 64 (1 RCT) ^1^ | ⨁◯◯◯ VERY LOW | We are uncertain about the effect on this outcome because the certainty of the evidence is very low. |
| Side effects: vomiting | 75 per 1,000 | **215 per 1,000** (58 to 788) | **RR 2.87** (0.77 to 10.50) | 64 (1 RCT) ^1^ | ⨁◯◯◯ VERY LOW | We are uncertain about the effect on this outcome because the certainty of the evidence is very low. |
| Satisfaction | 450 per 1,000 | **455 per 1,000** (243 to 846) | **RR 1.01** (0.54 to 1.88) | 64 (1 RCT) ^1^ | ⨁◯◯◯ VERY LOW | No direct evidence identified. |
| ***The risk in the intervention group** (and its 95% confidence interval) is based on the assumed risk in the comparison group and the **relative effect** of the intervention (and its 95% CI).   **CI:** Confidence interval; **RR:** Risk ratio. **Studies:** Blanchard 2005^1^ | | | | | | |

1. **Misoprostol 400 μg oral every 3 hours for 4 doses compared with 800 μg oral misoprostol every 6 hours for 2 doses**

| **Outcomes** | **Anticipated absolute effects^*^ (95% CI)** | | **Relative effect (95% CI)** | **№ of participants  (studies)** | **GRADE** | **Comments** |
| --- | --- | --- | --- | --- | --- | --- |
|  | **Risk with misoprostol 800 po q6 x 2** | **Risk with misoprostol 400 mcg po q 3x4** |  |  |  |  |
| Efficacy: ongoing pregnancy (Efficacy) | 167 per 1,000 | **292 per 1,000** (103 to 817) | **RR 1.75** (0.62 to 4.90) | 60 (1 RCT) ^1^ | ⨁◯◯◯ VERY LOW | We are uncertain about the effect on this outcome because the certainty of the evidence is very low. |
| Efficacy: completed without surgical intervention | 500 per 1,000 | **420 per 1,000** (220 to 795) | **RR 0.84** (0.44 to 1.59) | 60 (1 RCT) ^1^ | ⨁◯◯◯ VERY LOW | We are uncertain about the effect on this outcome because the certainty of the evidence is very low. |
| Safety: serious adverse events and complications | 0 per 1,000 | **0 per 1,000** (0 to 0) | not estimable | (0 studies) | - | No direct evidence identified |
| Expulsion time <24 hours | 0 per 1,000 | **0 per 1,000** (0 to 0) | not estimable | (0 studies) | - | No direct evidence identified |
| Side effects: bleeding | 0 per 1,000 | **0 per 1,000** (0 to 0) | not estimable | (0 studies) | - | No direct evidence identified |
| Side effects: pain | 958 per 1,000 | **958 per 1,000** (661 to 1,000) | **RR 1.00** (0.69 to 1.45) | 60 (1 RCT) ^1^ | ⨁◯◯◯ VERY LOW | We are uncertain about the effect on this outcome because the certainty of the evidence is very low. |
| Side effects: vomiting | 250 per 1,000 | **333 per 1,000** (140 to 780) | **RR 1.33** (0.56 to 3.12) | 60 (1 RCT) ^1^ | ⨁◯◯◯ VERY LOW | We are uncertain about the effect on this outcome because the certainty of the evidence is very low. |
| Satisfaction | 458 per 1,000 | **408 per 1,000** (211 to 788) | **RR 0.89** (0.46 to 1.72) | 60 (1 RCT) ^1^ | ⨁◯◯◯ VERY LOW | No direct evidence identified. |
| ***The risk in the intervention group** (and its 95% confidence interval) is based on the assumed risk in the comparison group and the **relative effect** of the intervention (and its 95% CI).   **CI:** Confidence interval; **RR:** Risk ratio. **Studies:** Blanchard 2005^1^ | | | | | | |

**Table S6. Comparison of medical and surgical management- Medical management with 800 μg vaginal misoprostol compared with surgical management**

| **Outcomes** | **Anticipated absolute effects^*^ (95% CI)** | | **Relative effect (95% CI)** | **№ of participants  (studies)** | **GRADE** | **Comments** |
| --- | --- | --- | --- | --- | --- | --- |
|  | **Risk with surgical management** | **Risk with 800 μg vaginal misoprostol** |  |  |  |  |
| Efficacy: ongoing pregnancy (Efficacy) | 0 per 1,000 | **0 per 1,000** (0 to 0) | **RR 6.70** (1.88 to 23.86) | 137 (1 RCT) ^1^ | ⨁◯◯◯ VERY LOW | We are uncertain about the effect on this outcome because the certainty of the evidence is very low. |
| Efficacy: completed without surgical intervention | 956 per 1,000 | **975 per 1,000** (851 to 1,000) | **RR 1.02** (0.89 to 1.17) | 137 (1 RCT) ^1^ | ⨁◯◯◯ VERY LOW | We are uncertain about the effect on this outcome because the certainty of the evidence is very low. |
| Safety: serious adverse events and complications | 15 per 1,000 | **5 per 1,000** (0 to 118) | **RR 0.33** (0.01 to 8.04) | 137 (1 RCT) ^1^ | ⨁◯◯◯ VERY LOW | We are uncertain about the effect on this outcome because the certainty of the evidence is very low. |
| Expulsion time <24 hours | 956 per 1,000 | **679 per 1,000** (497 to 927) | **RR 0.71** (0.52 to 0.97) | 137 (1 RCT) ^1^ | ⨁◯◯◯ VERY LOW | We are uncertain about the effect on this outcome because the certainty of the evidence is very low. |
| Side effects: bleeding | 0 per 1,000 | **0 per 1,000** (0 to 0) | **RR 6.60** (0.34 to 125.00) | 137 (1 RCT) ^1^ | ⨁◯◯◯ VERY LOW | We are uncertain about the effect on this outcome because the certainty of the evidence is very low. |
| Side effects: pain | 1,000 per 1,000 | **700 per 1,000** (510 to 950) | **RR 0.70** (0.51 to 0.95) | 137 (1 RCT) ^1^ | ⨁◯◯◯ VERY LOW | We are uncertain about the effect on this outcome because the certainty of the evidence is very low. |
| Side effects: vomiting | 29 per 1,000 | **56 per 1,000** (11 to 297) | **RR 1.91** (0.36 to 10.10) | 137 (1 RCT) ^1^ | ⨁◯◯◯ VERY LOW | We are uncertain about the effect on this outcome because the certainty of the evidence is very low. |
| Satisfaction | 0 per 1,000 | **0 per 1,000** (0 to 0) | not estimable | (0 studies) ^1^ | - | No direct evidence identified. |
| ***The risk in the intervention group** (and its 95% confidence interval) is based on the assumed risk in the comparison group and the **relative effect** of the intervention (and its 95% CI).   **CI:** Confidence interval; **RR:** Risk ratio. **Studies:** Prasad 2009^1^ | | | | | | |
